# Percutaneous coronary intervention using new-generation drug-eluting stents versus coronary arterial bypass grafting in stable patients with multi-vessel coronary artery disease: From the CREDO-Kyoto PCI/CABG registry Cohort-3

**DOI:** 10.1371/journal.pone.0267906

**Published:** 2022-09-29

**Authors:** Hiroki Watanabe, Ko Yamamoto, Hiroki Shiomi, Takeshi Morimoto, Eri Kato, Yukiko Matsumura, Kenji Nakatsuma, Yasuaki Takeji, Hidenori Yaku, Erika Yamamoto, Yugo Yamashita, Yusuke Yoshikawa, Masayuki Fuki, Kyohei Yamaji, Natsuhiko Ehara, Hiroki Sakamoto, Kazuaki Imada, Takeshi Tada, Ryoji Taniguchi, Ryusuke Nishikawa, Tomohisa Tada, Takashi Uegaito, Tatsuya Ogawa, Miho Yamada, Teruki Takeda, Hiroshi Eizawa, Nobushige Tamura, Keiichi Tambara, Satoru Suwa, Manabu Shirotani, Toshihiro Tamura, Moriaki Inoko, Junichiro Nishizawa, Masahiro Natsuaki, Hiroshi Sakai, Takashi Yamamoto, Naoki Kanemitsu, Nobuhisa Ohno, Katsuhisa Ishii, Akira Marui, Hiroshi Tsuneyoshi, Yasuhiko Terai, Shogo Nakayama, Kazuhiro Yamazaki, Mamoru Takahashi, Takashi Tamura, Jiro Esaki, Shinji Miki, Tomoya Onodera, Hiroshi Mabuchi, Yutaka Furukawa, Masaru Tanaka, Tatsuhiko Komiya, Yoshiharu Soga, Michiya Hanyu, Kenji Ando, Kazushige Kadota, Kenji Minatoya, Yoshihisa Nakagawa, Takeshi Kimura, On behalf the CREDO-Kyoto PCI/CABG Registry Cohort-3 Investigators

**Affiliations:** 1 Department of Cardiology, Japanese Red Cross Wakayama Medical Center, Wakayama, Japan; 2 Department of Cardiovascular Medicine, Kyoto University Graduate School of Medicine, Kyoto, Japan; 3 Department of Clinical Epidemiology, Hyogo College of Medicine, Nishinomiya, Japan; 4 Department of Cardiology, Mitsubishi Kyoto Hospital, Kyoto, Japan; 5 Department of Cardiology, Kokura Memorial Hospital, Kitakyushu, Japan; 6 Department of Cardiovascular Medicine, Kobe City Medical Center General Hospital, Kobe, Japan; 7 Department of Cardiology, Shizuoka General Hospital, Shizuoka, Japan; 8 Department of Cardiology, Kurashiki Central Hospital, Kurashiki, Japan; 9 Department of Cardiology, Hyogo Prefectural Amagasaki General Medical Center, Amagasaki, Japan; 10 Department of Cardiology, Kishiwada City Hospital, Kishiwada, Japan; 11 Department of Cardiovascular Surgery, Kishiwada City Hospital, Kishiwada, Japan; 12 Department of Cardiology, Hamamatsu Rosai Hospital, Hamamatsu, Japan; 13 Department of Cardiology, Koto Memorial Hospital, Higashiomi, Japan; 14 Department of Cardiology, Kobe City Nishi-Kobe Medical Center, Kobe, Japan; 15 Department of Cardiovascular Surgery, Kindai University Nara Hospital, Ikoma, Japan; 16 Department of Cardiovascular Surgery, Juntendo University Shizuoka Hospital, Izunokuni, Japan; 17 Department of Cardiology, Juntendo University Shizuoka Hospital, Izunokuni, Japan; 18 Department of Cardiology, Kindai University Nara Hospital, Ikoma, Japan; 19 Department of Cardiology, Tenri Hospital, Tenri, Japan; 20 Department of Cardiology, Kitano Hospital, Osaka, Japan; 21 Department of Cardiovascular Surgery, Hamamatsu Rosai Hospital, Hamamatsu, Japan; 22 Department of Cardiovascular Medicine, Saga University, Saga, Japan; 23 Department of Cardiology, Shiga University of Medical Science Hospital, Otsu, Japan; 24 Department of Cardiovascular Surgery, Japanese Red Cross Wakayama Medical Center, Wakayama, Japan; 25 Department of Cardiovascular Surgery, Hyogo Prefectural Amagasaki General Medical Center, Amagasaki, Japan; 26 Department of Cardiology, Kansai Denryoku Hospital, Osaka, Japan; 27 Department of Cardiovascular Surgery, Kokura Memorial Hospital, Kitakyushu, Japan; 28 Department of Cardiovascular Surgery, Shizuoka General Hospital, Shizuoka, Japan; 29 Department of Cardiovascular Surgery, Shizuoka City Shizuoka Hospital, Shizuoka, Japan; 30 Department of Cardiovascular Surgery, Osaka Red Cross Hospital, Osaka, Japan; 31 Department of Cardiovascular Surgery, Kyoto University Graduate School of Medicine, Kyoto, Japan; 32 Department of Cardiology, Shimabara Hospital, Kyoto, Japan; 33 Department of Cardiovascular Surgery, Mitsubishi Kyoto Hospital, Kyoto, Japan; 34 Department of Cardiology, Shizuoka City Shizuoka Hospital, Shizuoka, Japan; 35 Department of Cardiology, Osaka Red Cross Hospital, Osaka, Japan; 36 Department of Cardiovascular Surgery, Kurashiki Central Hospital, Kurashiki, Japan; 37 Department of Cardiovascular Surgery, Kitano Hospital, Osaka, Japan; Bern University Hospital, SWITZERLAND

## Abstract

**Aims:**

There is a scarcity of studies comparing percutaneous coronary intervention (PCI) using new-generation drug-eluting stents (DES) with coronary artery bypass grafting (CABG) in patients with multi-vessel coronary artery disease.

**Methods and results:**

The CREDO-Kyoto PCI/CABG registry Cohort-3 enrolled 14927 consecutive patients who underwent first coronary revascularization with PCI or isolated CABG between January 2011 and December 2013. The current study population consisted of 2464 patients who underwent multi-vessel coronary revascularization including revascularization of left anterior descending coronary artery (LAD) either with PCI using new-generation DES (N = 1565), or with CABG (N = 899). Patients in the PCI group were older and more often had severe frailty, but had less complex coronary anatomy, and less complete revascularization than those in the CABG group. Cumulative 5-year incidence of a composite of all-cause death, myocardial infarction or stroke was not significantly different between the 2 groups (25.0% versus 21.5%, P = 0.15). However, after adjusting confounders, the excess risk of PCI relative to CABG turned to be significant for the composite endpoint (HR 1.27, 95%CI 1.04–1.55, P = 0.02). PCI as compared with CABG was associated with comparable adjusted risk for all-cause death (HR 1.22, 95%CI 0.96–1.55, P = 0.11), and stroke (HR 1.17, 95%CI 0.79–1.73, P = 0.44), but with excess adjusted risk for myocardial infarction (HR 1.58, 95%CI 1.05–2.39, P = 0.03), and any coronary revascularization (HR 2.66, 95%CI 2.06–3.43, P<0.0001).

**Conclusions:**

In this observational study, PCI with new-generation DES as compared with CABG was associated with excess long-term risk for major cardiovascular events in patients who underwent multi-vessel coronary revascularization including LAD.

## Introduction

Randomized controlled trials (RCT) in the first-generation drug-eluting stent (DES) era have clearly demonstrated the superiority of coronary artery bypass grafting (CABG) over percutaneous coronary intervention (PCI) in patients with complex multi-vessel coronary artery disease (CAD) [1, 2]. In contrast, several studies comparing PCI using the new-generation DES with CABG were performed and their results were very similar in that there was no difference in all-cause mortality but a higher rate of myocardial infarction and repeat revascularization in patients treated by PCI [3–5]. Fundamentally, RCTs are the golden-standard way to evaluate treatment method but have inherent limitation due to their broad exclusion criteria. Moreover, unlike clinical pharmacological trials, the study comparing PCI and CABG could be strongly influenced by the technical expertise, which might be substantially different among each RCTs and among the local clinical practices. For example, the proportion of IVUS use in the PCI group was very low in the previous RCTs. In this sense, observational studies might have a complementary role in comparing clinical outcomes after PCI and CABG. We already reported the comparison of long-term outcomes after PCI and CABG in patients with triple-vessel CAD in a large Japanese observational database in the new-generation DES era [4]. However, the study also had limitation in that it included those patients who did not undergo multi-vessel coronary revascularization, those who did not receive revascularization of left anterior descending coronary artery (LAD), and those who were not treated with new-generation DES, although the current guidelines endorsed exclusive use of new-generation DES for PCI, and recommended CABG as a class I indication in patients who need multi-vessel revascularization including revascularization of LAD [6, 7]. Therefore, we sought to compare the long-term clinical outcomes in the patients who underwent multi-vessel coronary revascularization including revascularization of LAD either by isolated CABG or by PCI with exclusive use of new-generation DES in a large Japanese observational database in the new-generation DES era.

## Methods

The Coronary Revascularization Demonstrating Outcome Study in Kyoto (CREDO-Kyoto) PCI/CABG registry Cohort-3 is a physician-initiated, non-company sponsored, multi-center registry enrolling consecutive patients who underwent first coronary revascularization with PCI or isolated CABG without combined non-coronary surgery among 22 Japanese centers between January 2011 and December 2013 [4]. The relevant ethics committees in all the participating centers approved the study protocol (S1 Appendix). Because of the retrospective enrollment, written informed consents from the patients were waived; however, we excluded those patients who refused participation in the study when contacted for follow-up.

**Fig 1:**
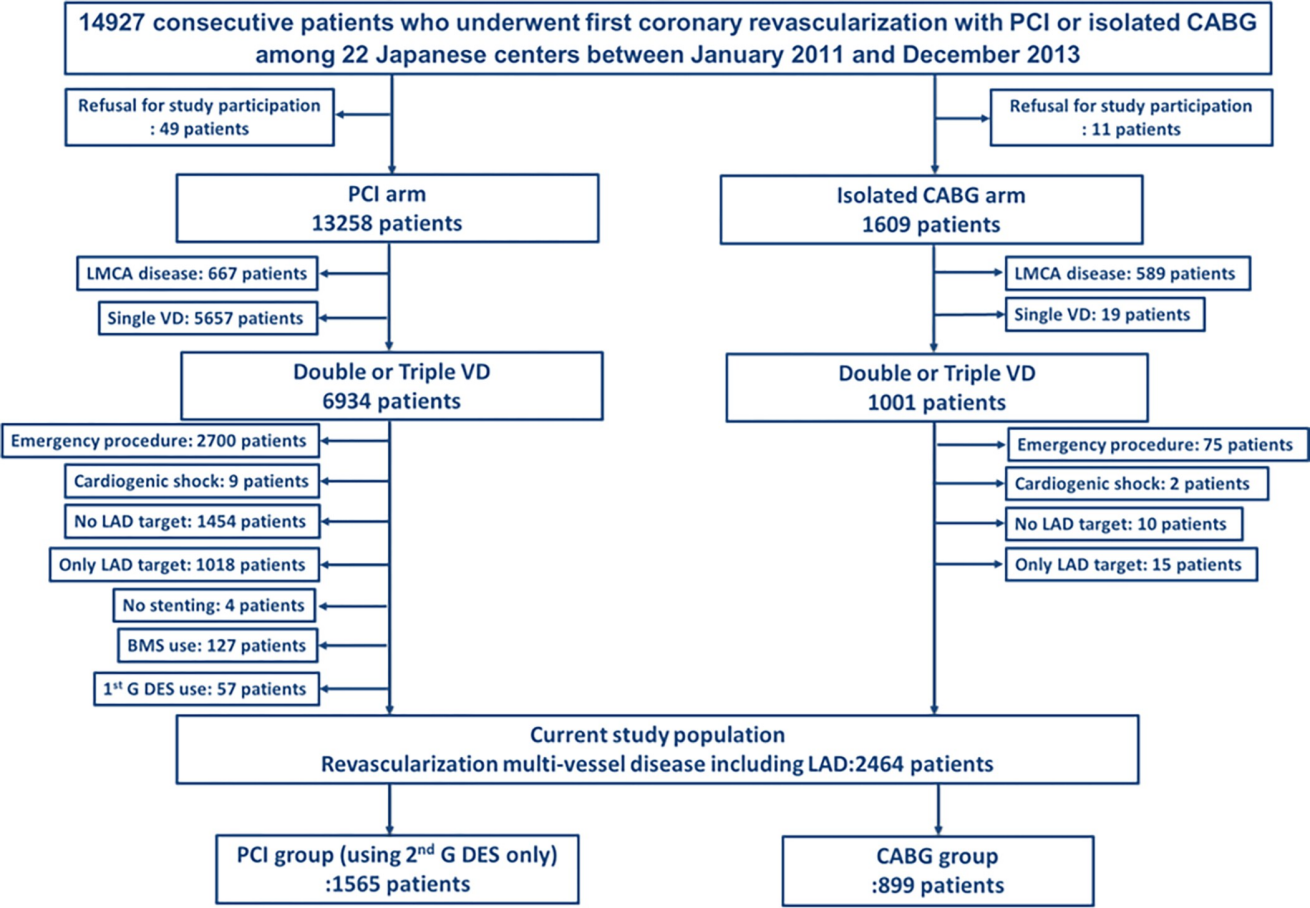
Study flowchart. BMS = bare-metal stent; CREDO-Kyoto PCI/CABG Registry Cohort-3 = Coronary Revascularization Demonstrating Outcome Study in Kyoto PCI/CABG registry; CABG = coronary artery bypass grafting; G-1 DES = first-generation drug-eluting stent; LAD = left anterior descending coronary artery; LMCA = left main coronary artery; PCI = percutaneous coronary intervention; G-2 DES = second-generation drug-eluting stent.

In the CREDO-Kyoto PCI/CABG registry Cohort-3, there were 14927 patients who underwent first coronary revascularization with PCI or isolated CABG (PCI: N = 13307, and CABG: N = 1620). In the present study, we excluded those patients who refused study participation (N = 60), those who had left main CAD (N = 1256), or single-vessel disease (N = 5657), those with emergency procedure (N = 2775), cardiogenic shock (N = 11), no LAD target (N = 1464), only LAD target (N = 1033), no stenting (N = 4), bare-metal stents use (N = 127) and first-generation DES use (N = 57). Finally, we identified 2464 patients who received multi-vessel coronary revascularization including revascularization of LAD either by isolated CABG (N = 899) or by PCI with exclusive use of new-generation DES (N = 1565) (Fig 1).

The primary outcome measure for the current analysis was a composite of all-cause death, myocardial infarction, or stroke. The secondary outcome measures included all-cause death, cardiovascular death, cardiac death, non-cardiovascular death, non-cardiac death, myocardial infarction, definite stent thrombosis or symptomatic graft occlusion, stroke, hospitalization for heart failure, major bleeding, target-vessel revascularization, ischemia-driven target-vessel revascularization, non-target-vessel revascularization, ischemia-driven non-target-vessel revascularization. any coronary revascularization, ischemia-driven any coronary revascularization, and a composite of all-cause death, myocardial infarction, stroke, or any coronary revascularization. Death was regarded as cardiac in origin unless obvious non-cardiac causes could be identified. Cardiovascular death included cardiac death, and other vascular death related to stroke, renal disease, and vascular disease. Death of unknown cause and any death during the index hospitalization for coronary revascularization were regarded as cardiac death. Myocardial infarction (MI) was categorized into periprocedural and spontaneous MI. We defined “emergency procedure” as those procedures for ACS patients that were performed immediately after clinical presentation. The definition of proximal LAD lesion was the lesion location in segment 6 and/or segment 7 by the American Heart Association (AHA) classification [8]. Severe frailty was regarded as present when the inability to perform usual activities of daily living was documented in the hospital charts. The staged PCI procedures were defined as scheduled procedures within 90 days after the index procedure, which were not regarded as the follow-up event, but regarded as the part of the index PCI procedure. The registry included both types of patients who underwent single-session multi-vessel PCI procedure, and who underwent staged multi-vessel PCI procedures.” Finally, definition of complete revascularization was the anatomical complete revascularization of all the stenotic lesions. Definitions of other baseline characteristics and outcome measures were previously described [4].

Clinical, angiographic, and procedural data were collected from hospital charts or hospital databases according to the pre-specified definitions by the experienced clinical research coordinators from an independent clinical research organization (Research Institute for Production Development, Kyoto, Japan) (S2 Appendix). Follow-up data were collected from the hospital charts and/or obtained by contact with patients, their relatives or physicians in charge between January 2018 and December 2019. Follow-up was regarded as completed, if follow-up data beyond July 1, 2017 were obtained. The clinical event committee adjudicated those events such as death, myocardial infarction, definite stent thrombosis or symptomatic graft occlusion, stroke, and major bleeding. Coronary anatomic complexity was evaluated in patients with triple-vessel and left main CAD according to the SYNTAX score, which was evaluated by the experienced cardiologists (S3 Appendix).

Categorical variables were presented as number and percentage, and compared with the chi-square test. Continuous variables were expressed as mean ± standard deviation or median and interquartile range. Continuous variables were compared with the Student’s t test or Wilcoxon rank sum test based on their distributions. Cumulative incidences of the outcome measures were estimated by the Kaplan-Meier method, and the differences were assessed with the log-rank test. The risks of PCI relative to CABG for the outcome measures were estimated in the Cox proportional hazard models adjusting for the 27 clinically relevant factors listed in Table 1, and were expressed as hazard ratios (HRs) and their 95% confidence intervals (CIs). We did not utilize the variables selection procedures such as stepwise selection. Continuous variables were dichotomized by clinically meaningful reference values to make proportional hazard assumptions robust and consistent with our previous reports. To avoid over-fitting, we constructed a parsimonious model for hemorrhagic stroke with 8 risk-adjusting variables including advanced age (> = 75 years), men, diabetes, heart failure, prior myocardial infarction, prior stroke, end-stage renal disease (estimated glomerular filtration rate [eGFR] <30 mL/min/1.73m^2^ or hemodialysis), and severe frailty, because the number of patients with event was <100 for this outcome measure. Furthermore, we did not perform a multivariable analysis for the outcome measures with <30 patients with event. We performed subgroup analyses for the primary outcome measure stratified by age (> = or < 75 years), sex, diabetes, heart failure, end-stage renal disease (eGFR <30 mL/min/1.73m^2^ or hemodialysis), extent of CAD (double-vessel or triple-vessel disease), and complete revascularization (complete or incomplete revascularization). Furthermore, we showed clinical outcomes of two subgroups; patients with two-vessel disease who received coronary revascularization including revascularization of LAD, and patients with multi-vessel coronary revascularization including revascularization of proximal LAD (S1 and S2 Tables and S1 and S2 Figs). As a sensitivity analysis, we also conducted the propensity-score matching analysis (S1 Method and S3 and S4 Tables). Finally, we divided patients in the PCI group according to the completeness of revascularization and showed Kaplan-Meier event curves for target-vessel revascularization and non-target-vessel revascularization in the two groups (S2 Method and S3 Fig). Statistical analyses were performed with JMP 14.0 software (SAS Institute, Inc., Cary, North California). All statistical analyses were 2 tailed, and P values of <0.05 were considered statistically significant.

**Table 1:** Baseline characteristics and medications during the index hospitalization: PCI group versus CABG group.

Variables	PCI group	CABG group	P value
	N = 1565	N = 899	
**Clinical characteristics**			
Age	70.2±10.3	68.6±9.6	<0.0001
*> = 75years	566(36%)	266(30%)	0.0008
*Male	1112(71%)	699(78%)	0.0003
Body mass index (kg/m^2^)	24.0±3.7 (N = 1558)	23.8±3.5 (N = 898)	0.10
*<25.0kg/m^2^	1007(64%)	597(66%)	0.30
*Hypertension	1351(86%)	774(86%)	0.87
*Diabetes mellitus	740(47%)	478(53%)	0.005
requiring insulin therapy	175(11%)	189(21%)	< .0001
Current smoking	326(21%)	159(18%)	0.06
*Heart failure	334(21%)	231(26%)	0.01
History of heart failure	117(7.5%)	167(19%)	< .0001
Current heart failure	263(17%)	102(11%)	< .0001
Clinical presentation			0.001
Stable angina	940(60%)	567(63%)	
Unstable angina	19(1.2%)	11(1.2%)	
AMI	70(4.5%)	18(2.0%)	
Silent myocardial ischemia	131(8.4%)	99(11%)	
Old myocardial infarction without angina	144(9.2%)	73(8.1%)	
Coronary stenosis without documentation of myocardial ischemia	261(16.7%)	131(8.4%)	
LVEF	59±14	58±14	0.14
LVEF < = 40%	157/1408(11%)	111/863(13%)	0.22
Mitral regurgitation grade> = 3/4	96/1413(6.8%)	70/864(8.1%)	0.25
*Previous myocardial infarction	303(19%)	232(26%)	0.0002
*Previous symptomatic stroke	230(15%)	158(18%)	0.06
*Peripheral vascular disease	164(10%)	128(14%)	0.006
eGFR<30mL/min/1.73m^2^ or hemodialysis	139(8.9%)	132(15%)	<0.0001
*eGFR<30mL/min/1.73m^2^ without hemodialysis	51(3.3%)	58(6.5%)	0.0003
*Hemodialysis	88(5.6%)	74(8.2%)	0.01
*Atrial fibrillation	129(8.2%)	69(7.7%)	0.62
*Anemia (hemoglobin<11.0g/dL)	213(14%)	158(18%)	0.009
*Thrombocytopenia (Platelet < 100*10⁹/L)	24(1.5%)	24(2.7%)	0.053
*Chronic obstructive pulmonary disease	50(3.2%)	46(5.1%)	0.02
*Liver cirrhosis	43(2.8%)	28(3.1%)	0.60
Malignancy	179(11%)	103(11%)	0.99
*Active malignancy	20(1.3%)	20(2.2%)	0.08
*Severe fraility	58(3.7%)	14(1.6%)	0.001
**Baseline medications**			
Antiplatelet therapy			
Thienopyridine	1561(100%)	194(22%)	<0.0001
Ticlopidine	34(2.2%)	14(1.6%)	
Clopidogrel	1521(97%)	180(20%)	
Unknown	6(0.4%)	0	
Aspirin	1558(100%)	884(98%)	0.003
Cilostazole	45(2.9%)	27(3.0%)	0.86
Other medications			
*Statin	1206(77%)	586(65%)	<0.0001
High-intensity statin	23(1.5%)	5(0.6%)	0.03
*ACE-I/ARB	993(63%)	276(31%)	<0.0001
*β blocker	532(34%)	508(57%)	<0.0001
Nitrate	387(25%)	112(12%)	<0.0001
*Calcium channel blocker	787(50%)	340(38%)	<0.0001
Nicorandil	240(15%)	333(37%)	<0.0001
*Oral anticoagulants	130(8.3%)	483(54%)	<0.0001
Warfarin	114(7.3%)	477(53%)	<0.0001
DOAC	16(1.0%)	6(0.7%)	0.36
*Proton pump inhibitor or histamine type-2 receptor blocker	1126(72%)	831(92%)	<0.0001
Proton pump inhibitor	960(61%)	758(84%)	<0.0001
Histamine type-2 receptor blocker	174(11%)	75(8.3%)	0.03

Categorical variables are expressed as number (%) unless otherwise indicated. Continuous variables are shown as mean ± SD.

* Potential risk-adjusting variables selected for multivariable analysis.

ACE-I/ARB = angiotensin converting enzyme inhibitor/angiotensin receptor blocker; CABG = coronary artery bypass grafting; DOAC = direct oral anticoagulants; eGFR = estimated glomerular filtration rate; LVEF = left ventricular ejection fraction; PCI = percutaneous coronary intervention.

## Results

The current study population consisted of 1565 patients (64%) who received PCI with the second-generation DES and 899 patients (36%) who underwent CABG. The patients in the PCI group were older and more often had current heart failure, and severe frailty than those in the CABG group, while the patients in the CABG group had higher prevalence of men, diabetes mellitus, prior heart failure, prior myocardial infarction, peripheral vascular disease, eGFR <30 mL/min/1.73m^2^ without hemodialysis, and hemodialysis (Table 1). As for the angiographic and procedural characteristics, the CABG group had greater number of target lesions or anastomoses and higher coronary anatomical complexity as indicated by the greater number of targets for chronic total occlusion and higher prevalence of triple-vessel disease. Intracoronary imaging, predominantly intravascular ultrasound (IVUS), was used in 87% of patients in the PCI group. Complete revascularization was more frequently achieved in the CABG group than in the PCI group (84% versus 68%, P<0.0001); All the patients with two-vessel disease underwent complete revascularization in both groups, while complete revascularization was achieved much more frequently in the CABG group than in the PCI group (81% versus 39%, P<0.0001) (Table 2). In terms of baseline medications, statins and angiotensin converting enzyme inhibitor/angiotensin II receptor blockers were more often prescribed in the PCI group than in the CABG group, while β blockers were more often prescribed in the CABG group than in the PCI group. The prevalence of high-intensity statins therapy was very low in both groups. Thienopyridines were prescribed in 194 patients (22%) in the CABG group, but were discontinued before the index procedure in 118 patients. Oral anticoagulants were more often prescribed in the CABG group than in the PCI group, mainly because atrial fibrillation was newly identified after the index procedure in 187 patients (21%) in the CABG group (Table 1).

**Table 2:** Angiographic and procedural characteristics: PCI group versus CABG group.

Variables	PCI group	CABG group	P value
	N = 1565	N = 899	
Three-vessel disease	814(52%)	764(85%)	<0.0001
Chronic total occlusion (target or non-target)	495(32%)	467(52%)	<0.0001
Location of CTO (target and non-target)			
LAD	198(13%)	194(22%)	<0.0001
LCX	163(10%)	149(17%)	<0.0001
RCA	229(15%)	275(31%)	<0.0001
Number of target lesion or anastomoses	2.5±0.7	3.4±0.9	<0.0001
SYNTAX score	25±8.5(N = 776)	29±8.0(N = 607)	<0.0001
Low (<23)	329/776(42%)	128/607(21%)	<0.0001
Intermediate (23–32)	315/776(41%)	277/607(46%)	0.06
High (> = 33)	132/776(17%)	202/607(33%)	<0.0001
Target vessel or anastomoses			
*Proximal LAD	1508(96%)	854(95%)	0.11
LCX	911(58%)	759(84%)	<0.0001
RCA	971(62%)	762(85%)	<0.0001
*Target CTO vessel	359(23%)	444(49%)	<0.0001
Total number of stents	3(2–4)	-	
Total stent length (mm)	70(48–97)	-	
Type of DES			
Everolimus-eluting stent (XIENCE™) use	972(62%)	-	
Everolimus-eluting stent (PROMUS™) use	429(27%)	-	
Biolimus-eluting stent (NOBORI™) use	511(33%)	-	
Zotarolimus-eluting stent (RESOLUTE™) use	136(8.7%)	-	
Zotarolimus-eluting stent (ENDEAVOR™) use	18(1.2%)	-	
IVUS or OCT use	1355(87%)	-	
IVUS use	1352(86%)	-	
FFR evaluation before treatment	139(8.9%)	0	<0.0001
Number of CTO lesions (target and non-target)			<0.0001
	0.6±0.4	0.8±0.7	
	0(0–1)	1(0–1)	
Number of CTO target lesions			<0.0001
	0.5±0.3	0.6±0.7	
	0(0–1)	0(0–1)	
Location of target CTO vessels			
LAD	157(10%)	194(22%)	<0.0001
LCX	95(6.1%)	132(15%)	<0.0001
RCA	165(11%)	246(27%)	<0.0001
Successful CTO PCI	313/359(87%)	-	
Bifurcated lesion	941(60%)	-	
Side-branch stenting	145(9.3%)	-	
Staged PCI	1000(64%)	-	
Complete revascularization	1068(68%)	757(84%)	<0.0001
Two-vessel disease (N = 751)	751/751(100%)	135/135(100%)	-
Three-vessel disease (N = 814)	317/814(39%)	622/764(81%)	<0.0001
Internal thoracic artery graft use	-	881(98%)	
Off-pump surgery	-	527(59%)	

* Potential risk-adjusting variables selected for multivariable analysis.

** SYNTAX score was calculated only among patients with triple-vessel disease.

*** The rate of successful CTO-PCI was calculated among patients with target of CTO.

CABG = coronary artery bypass grafting; CTO = chronic total occlusion; DES = drug-eluting stent; IVUS = intravascular ultrasound; LAD = left anterior descending coronary artery; LCX = left circumflex coronary artery; OCT = optical coherence tomography; PCI = percutaneous coronary intervention; RCA = right coronary artery; SYNTAX = SYNergy between percutaneous coronary intervention with TAXus and cardiac surgery.

Median follow-up duration was 5.8 (interquartile range: 4.7–8.2) years, and complete 1-, 3-, 5-year clinical follow-up data were obtained in 97.0%, 91.7%, and 86.3% of patients, respectively without differences between the PCI and the CABG groups (97.2% versus 96.8%, 91.3% versus 92.6%, and 85.4% versus 87.8%, respectively).

**Fig 2:**
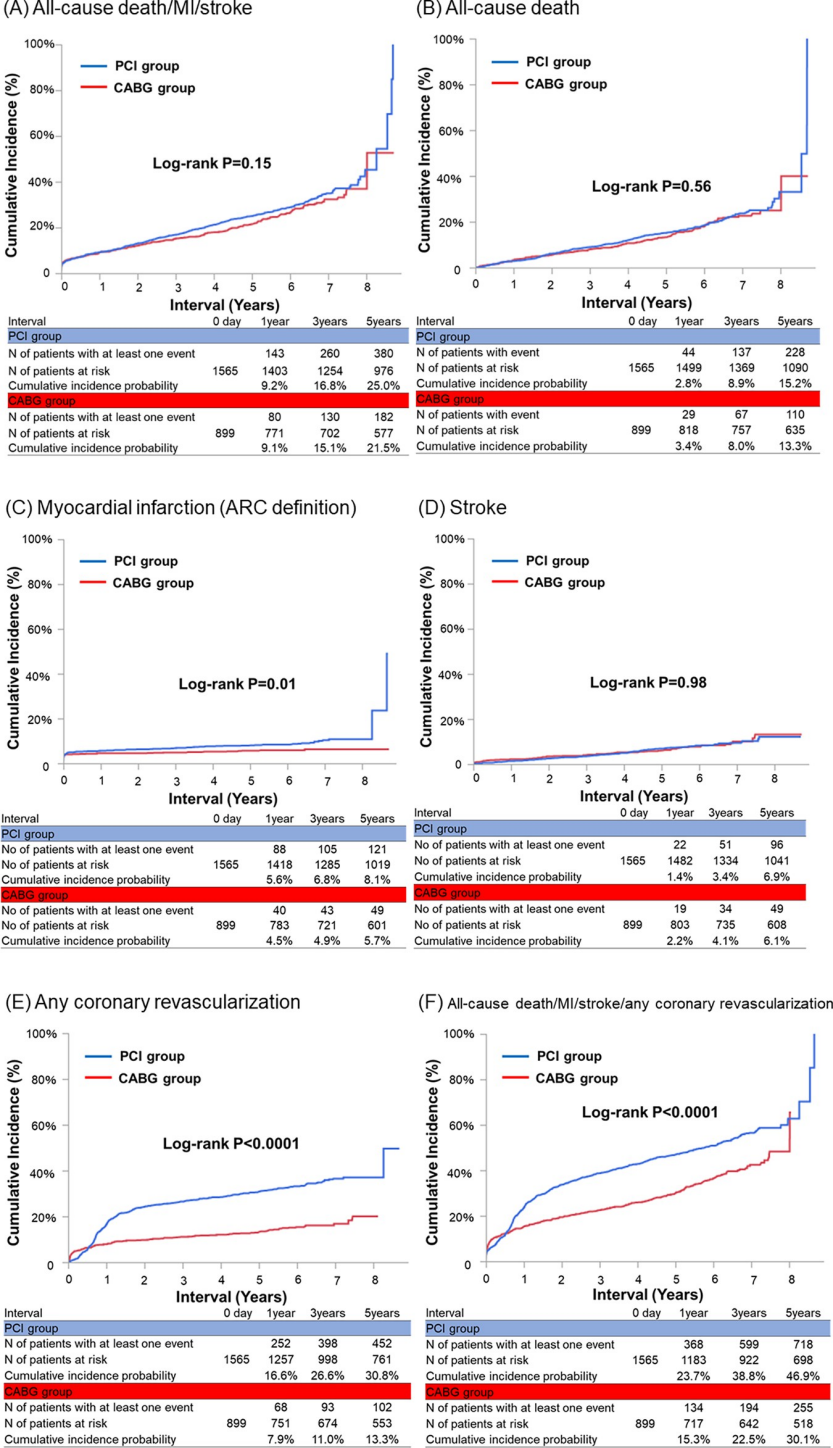
Crude kaplan-meier curves for the cumulative incidence of (A) all-cause death/MI/stroke, (B) all-cause death, (C) any coronary revascularization, and (D) all-cause death/MI/stroke/any coronary revascularization. PCI = percutaneous coronary intervention; CABG = coronary artery bypass grafting; MI = myocardial infarction.

The cumulative 5-year incidence of the primary outcome measure (a composite of death, myocardial infarction, or stroke) was not significantly different between the PCI and the CABG groups (25.0% versus 21.5%, log-rank P = 0.15) (Fig 2). However, after adjusting the confounders, the excess risk of PCI relative to CABG turned significant for the primary outcome measure (HR 1.27, 95%CI 1.04–1.55, P = 0.02) (Table 3). As for the individual component of the composite endpoint, the cumulative 5-year incidences of all-cause death and stroke were not significantly different between the 2 groups (15.2% versus 13.3%, log-rank P = 0.56, and 6.9% versus 6.1%, log-rank P = 0.98), whereas the cumulative 5-year incidence of myocardial infarction was significantly higher in the PCI group than that in the CABG group (8.1% versus 5.7%, log-rank P = 0.01) (Fig 2). Even after adjusting the confounders, the risks of PCI relative to CABG remained insignificant for all-cause death and stroke, while the excess risk of PCI relative to CABG remained significant for myocardial infarction (Table 3). PCI as compared with CABG was associated with substantially higher adjusted risk for target-vessel revascularization, non-target-vessel revascularization, any coronary revascularization, and a composite of all-cause death, myocardial infarction, stroke, or any coronary revascularization (Table 3). The higher risk of any coronary revascularization in the PCI group was related to TVR and also related to non-TVR.

**Table 3:** Clinical outcomes: PCI group versus CABG group.

Variables				PCI group	CABG group	Crude HR	P value	Adjusted HR	P value
				N of patients with events	N of patients with events				
				(Cumulative incidence)	(Cumulative incidence)	(95%CI)		(95%CI)	
				N = 1565	N = 899				
**Primary outcome measure**									
	**A composite of death, MI, or stroke**			475 (25.0%)	236 (21.5%)	1.12	0.15	1.27	0.02
						(0.96–1.31)		(1.04–1.55)	
**Secondary outcome measures**									
	**All-cause death**			305 (15.2%)	158 (13.3%)	1.06	0.56	1.22	0.11
						(0.87–1.29)		(0.96–1.55)	
		**Cardiovascular death**		149 (7.5%)	92 (8.5%)	0.89	0.38	1.09	0.60
						(0.69–1.16)		(0.79–1.52)	
		**Cardiac death**		108 (5.4%)	65 (6.6%)	0.91	0.56	1.15	0.48
						(0.67–1.24)		(0.78–1.69)	
			**Sudden cardiac death**	28 (1.6%)	20 (2.2%)	0.77	0.38	0.96	0.88
						(0.44–1.39)		(0.53–1.71)	
		**Non-cardiovascular death**		156 (8.3%)	66 (5.3%)	1.29	0.07	1.38	0.08
						(0.98–1.74)		(0.96–1.98)	
		**Non-cardiac death**		197 (10.3%)	93 (7.2%)	1.16	0.23	1.28	0.12
						(0.91–1.49)		(0.94–1.75)	
	**Myocardial infarction**								
		**ARC definition**		136 (8.1%)	51 (5.7%)	1.49	0.01	1.58	0.03
						(1.08–2.06)		(1.05–2.39)	
			**Periprocedural MI**	85 (5.4%)	35 (3.9%)	1.38	0.10	1.56	0.09
						(0.94–2.07)		(0.93–2.60)	
			**Spontaneous MI**	51 (2.8%)	16 (1.6%)	1.72	0.049	2.09	0.01
						(1.00–3.12)		(1.18–3.70)	
		**ARTS definition**		96 (5.5%)	26 (3.0%)	2.06	0.0005	2.24	0.004
						(1.35–3.23)		(1.30–3.85)	
	**Definite stent thrombosis or symptomatic graft occlusion**			10 (0.7%)	9 (1.2%)	0.62	0.30	NA	NA
						(0.25–1.56)		NA	
	**Stroke**			118 (6.9%)	65 (6.1%)	1.00	0.98	1.17	0.44
						(0.74–1.36)		(0.79–1.73)	
		**Ischemic stroke**		92 (5.2%)	51 (4.7%)	1.00	0.99	1.15	0.55
						(0.71–1.41)		(0.73–1.79)	
		**Hemorrhagic stroke**		33 (2.1%)	17 (1.7%)	1.08	0.80	1.09	0.77
						(0.61–1.98)		(0.60–1.98)	
		**Major stroke**		87 (5.1%)	49 (4.9%)	0.99	0.94	1.17	0.50
						(0.70–1.41)		(0.74–1.84)	
	**Hospitalization for heart failure**			155 (9.4%)	101 (10.5%)	0.82	0.14	1.00	1.00
						(0.64–1.06)		(0.73–1.37)	
	**Major bleeding**								
		**BARC type 3,4, or 5**		246 (15.2%)	340 (36.7%)	0.35	<0.0001	0.37	<0.0001
						(0.29–0.41)		(0.30–0.45)	
			**In-hospital bleeding**	39 (2.5%)	271 (30.2%)	0.08	<0.0001	0.09	<0.0001
						(0.06–0.11)		(0.06–0.13)	
			**Out-of-hospital bleeding**	207 (12.7%)	69 (6.7%)	1.69	0.0002	1.96	0.0001
						(1.28–2.21)		(1.39–2.77)	
		**BARC type 3**		222 (13.8%)	127 (13.4%)	0.97	0.76	1.05	0.71
						(0.78–1.20)		(0.80–1.39)	
		**BARC type 4**		12 (0.7%)	203 (22.5%)	0.03	<0.0001	0.04	<0.0001
						(0.02–0.06)		(0.02–0.07)	
		**BARC type 5**		12 (0.7%)	10 (1.1%)	0.66	0.34	NA	NA
						(0.29–1.57)		NA	
		**GUSTO moderate or severe**		203 (12.5%)	558 (61.6%)	0.16	<0.0001	0.16	<0.0001
						(0.13–0.18)		(0.13–0.20)	
			**In-hospital bleeding**	22 (1.4%)	536 (59.6%)	0.02	<0.0001	0.02	<0.0001
						(0.01–0.03)		(0.01–0.04)	
			**Out-of-hospital bleeding**	181 (11.0%)	22 (2.0%)	4.71	<0.0001	6.51	<0.0001
						(3.10–7.54)		(3.85–11.0)	
		**GUSTO severe**		115 (7.0%)	104 (11.2%)	0.59	0.0001	0.58	0.001
						(0.45–0.77)		(0.41–0.80)	
	**Target-vessel revascularization**			409 (25.3%)	112 (12.1%)	2.15	<0.0001	2.43	<0.0001
						(1.75–2.66)		(1.85–3.19)	
		**Ischemia-driven target-vessel revascularization**		202 (11.8%)	67 (7.0%)	1.70	<0.0001	1.54	0.02
						(1.30–2.26)		(1.09–2.18)	
	**Non-target-vessel revascularization**			172 (11.0%)	17 (1.7%)	5.82	<0.0001	6.05	<0.0001
						(3.65–9.94)		(3.38–10.8)	
		**Ischemia-driven non-target-vessel revascularization**		84 (5.3%)	10 (0.9%)	4.70	<0.0001	5.50	<0.0001
						(2.56–9.66)		(2,83–10.7)	
	**Any coronary revascularization**			491 (30.8%)	125 (13.1%)	2.37	<0.0001	2.66	<0.0001
						(1.96–2.90)		(2.06–3.43)	
		**Ischemia-driven any coronary revascularization**		240 (14.4%)	74 (7.5%)	1.84	<0.0001	1.56	0.008
						(1.43–2.41)		(1.12–2.16)	
	**A composite of death, MI, stroke, or any coronary revascularization**			809 (46.9%)	319 (30.1%)	1.58	<0.0001	1.70	<0.0001
						(1.39–1.80)		(1.43–2.01)	

Number of patients with event was counted until the end of follow-up. Cumulative incidence was estimated by the Kaplan-Meier method, and indicated at 5-year. HRs with 95% CIs of the PCI group relative to the CABG group for the outcome measures were estimated throughout the entire follow-up period by the Cox proportional hazard models. As the number of patients with event for hemorrhagic stroke was <100, we selected a parsimonious model with 8 risk-adjusting variables (age> = 75, men, diabetes mellitus, heart failure, prior myocardial infarction, prior stroke, eGFR <30 mL/min/1.73m^2^ or hemodialysis, and severe frailty). For the outcome measures with the number of patients with event <30, we did not perform a multivariable analysis.

*Myocardial infarction as a component of the composite outcome measure was adjudicated according to the ARC definition.

ARC = Academic Research Consortium; ARTS = Arterial Revascularization Therapy Study; BARC = Bleeding Academic Research Consortium; CABG = coronary artery bypass grafting; CI = confidence interval; PCI = percutaneous coronary intervention; CABG = coronary artery bypass grafting; eGFR = estimated glomerular filtration rate; HR = hazard ratio; CI = confidence interval; GUSTO = Global Utilization of Streptokinase and Tissue Plasminogen Activator for Occluded Coronary Arteries; HR = hazard ratio; MI = myocardial infarction; NA = not assessed; PCI = percutaneous coronary intervention.

In the subgroup analyses, there was no significant interaction between the subgroup factors and the risks of PCI relative to CABG for the primary outcome measure (Table 4).

**Table 4:** Subgroup analyses for the primary outcome measure (death/MI/stroke).

		PCI group	CABG group	Crude	P value	Adjusted	P value	P for interaction
		No. of patients with event/No. of patients at risk		HR		HR		
		(Cumulative incidence)	(Cumulative incidence)	(95%CI)		(95%CI)		
		N = 1565	N = 899					
**Age**								
	**> = 75 years**	**188/566 (34.6%)**	**71/266 (28.7%)**	**1.20(0.94–1.55)**	**0.14**	**1.24(0.96–1.60)**	**0.08**	**0.28**
	**<75 years**	**192/999 (19.7%)**	**111/633 (18.6%)**	**1.00(0.82–1.23)**	**0.96**	**1.36(0.97–1.91)**	**0.10**	
**Sex**								
	**Men**	**278/1112 (25.7%)**	**145/699 (22.2%)**	**1.09(0.91–1.30)**	**0.34**	**1.20(0.96–1.51)**	**0.11**	**0.42**
	**Women**	**102/453 (23.4%)**	**37/200 (19.4%)**	**1.27(0.91–1.80)**	**0.16**	**1.49(0.94–2.36)**	**0.09**	
**Diabetic status**								
	**YES**	**183/740 (25.5%)**	**99/478 (22.3%)**	**1.10(0.89–1.37)**	**0.38**	**1.30(0.96–1.76)**	**0.08**	**0.82**
	**No**	**197/825 (24.6%)**	**83/421 (20.7%)**	**1.14(0.91–1.44)**	**0.25**	**1.28(0.97–1.70)**	**0.08**	
**Heart failure**								
	**YES**	**120/334 (38.0%)**	**69/231 (31.8%)**	**1.20(0.92–1.58)**	**0.18**	**1.31(0.95–1.82)**	**0.10**	**0.83**
	**No**	**260/1231 (21.6%)**	**113/668 (18.0%)**	**1.15(0.95–1.39)**	**0.16**	**1.25(0.96–1.62)**	**0.10**	
**eGFR<30mL/min/1.73m^2^ or hemodialysis**								
	**YES**	**67/139 (50.9%)**	**49/132 (39.9%)**	**1.30(0.93–1.81)**	**0.13**	**1.30(1.02–1.65)**	**0.48**	**0.53**
	**No**	**313/1426 (22.6%)**	**133/767 (18.4%)**	**1.18(0.99–1.41)**	**0.07**	**1.16(0.78–1.74)**	**0.03**	
**Extent of coronary artery disease**								
	**Double-vessel disease**	**159/751 (21.8%)**	**24/135 (18.9%)**	**1.04(0.72–1.55)**	**0.83**	**1.45(0.91–2.32)**	**0.12**	**0.32**
	**Triple-vessel disease**	**221/814 (28.0%)**	**158/764 (22.0%)**	**1.30(1.09–1.56)**	**0.004**	**1.36(1.08–1.71)**	**0.01**	
**Complete revascularization**								
	**YES**	**283/1068 (22.3%)**	**182/757 (19.4%)**	**1.05(0.87–1.26)**	**0.63**	**1.17(0.91–1.50)**	**0.22**	**0.89**
	**No**	**192/497 (30.9%)**	**54/142 (32.4%)**	**1.02(0.76–1.39)**	**0.91**	**1.37(0.95–1.96)**	**0.09**	

Number of patients with event was counted until the end of follow-up. Cumulative 5-year incidence was estimated by the Kaplan-Meier method, and indicated at 5-year. HRs with 95% CIs of the PCI group relative to the CABG group for the primary outcome measure (death/MI/stroke) were estimated throughout the entire follow-up period by the Cox proportional hazard models.

CABG = coronary artery bypass grafting; CI = confidence interval; eGFR = estimated glomerular filtration rate; HR = hazard ratio; PCI = percutaneous coronary intervention.

In patients with two-vessel disease who underwent multi-vessel revascularization including LAD, the 5-year cumulative incidence of the primary outcome measure was not significantly different between the 2 groups and the risk of PCI relative to CABG remained insignificant for the primary outcome measure after adjusting for the confounders (21.8% versus 18.9%, log-rank P = 0.83 and HR 1.20, 95%CI 0.80–1.80, P = 0.38) (S1 Table and S1 Fig). In patients with multi-vessel coronary revascularization including revascularization of proximal LAD, the 5- year cumulative incidence was not significantly different between the 2 groups, and there was a numerically excess risk of PCI relative to CABG for the primary outcome measure after adjusting for the confounders (25.0% versus 21.9%, log-rank P = 0.28 and HR 1.22, 95%CI 0.99–1.49, P = 0.06) (S2 Table and S2 Fig).

After propensity-score matching in the sensitivity analysis, baseline characteristics of the PCI and the CABG groups were much more comparable than those in the entire population (S3 Table). The results in the propensity-score matching analyses were fully consistent with the results in the main analyses (S4 Table).

While the cumulative 5-year incidence of target-vessel revascularization was not significantly different between patients with complete revascularization (CR) and with incomplete revascularization (ICR) (24.3% versus 27.4%, log-rank P = 0.32), the cumulative 5-year incidence of non-target-vessel revascularization in patients with CR was significantly higher than that in those with ICR (6.2% versus 21.5%, log-rank P<0.0001) (S4 Fig).

## Discussion

The main findings in the current study were as follows; (1) PCI with exclusive use of the newer-generation DES was associated with a higher long-term risk for a composite of all-cause death, myocardial infarction, or stroke in patients who underwent multi-vessel coronary revascularization including LAD; (2) PCI as compared with CABG was associated with a comparable long-term risk for all-cause death and stroke, but was associated with a significantly higher long-term risk for myocardial infarction and any coronary revascularization.

In the new-generation DES era, there was only one moderate sized RCT comparing PCI with CABG, although RCTs in the first-generation DES era have clearly demonstrated benefit of CABG over PCI in patients with complex multi-vessel CAD in terms of survival, myocardial infarction, and repeat revascularization [1, 2]. The BEST (Randomized Comparison of Coronary Artery Bypass Surgery and Everolimus-Eluting Stent Implantation in the Treatment of Patients with Multivessel Coronary Artery Disease) trial was an only RCT conducted in the new-generation DES era, which compared PCI using everolimus-eluting stents with CABG in patients with multi-vessel CAD [5]. The trial was prematurely terminated after enrollment of 880 patients as opposed to the planned enrollment of 1776 patients. The cumulative 5-year incidence of the primary endpoint of a composite of death, myocardial infarction, or target-vessel revascularization was significantly higher in the PCI group than in the CABG group, although there was no difference in long-term mortality between the 2 groups. The results of the present analysis were in line with the BEST trial results. In the present study, PCI compared with CABG was more often selected for multi-vessel coronary revascularization including LAD. It was reassuring that we did not find significant difference in long-term mortality with a strategy favoring PCI even in patients who needed multi-vessel coronary revascularization including LAD. However, the excess long-term risk of PCI relative to CABG remained significant for myocardial infarction and any coronary revascularization, despite exclusive use of new-generation DES and very high prevalence of intracoronary imaging use. One of the reasons for the higher risk of PCI relative to CABG for myocardial infarction and any coronary revascularization might be the higher revascularization completeness in patients with three-vessel disease treated by CABG than in those treated by PCI. Compared with the previous report from the CREDO-Kyoto Cohort-2 analyzing the population with identical inclusion criteria, the present study suggested only small improvement in the cumulative 5-year incidence of a composite of death, myocardial infarction, stroke or any coronary revascularization (51.2% and 46.9%) despite introduction of new-generation DES and some improvement in medical therapy [9].

SYNTAX-Ⅱ study was initiated in 2014, where clinical outcomes of PCI patients treated with the SYNTAX-Ⅱstrategy were compared with the historical PCI (SYNTAX-Ⅰ PCI) and CABG (SYNTAX-Ⅰ CABG) population matched on the basis of SYNTAX score Ⅱ. The SYNTAX-Ⅱstrategy included use of a new-generation DES (the SYNERGY™ DES; Boston Scientific, Marlborough, MA, USA), target lesion selection based on physiological evaluation by instantaneous wave-free ratio (IFR) or fractional flow reserve (FFR) measurement, stent optimization by intravascular ultrasound guidance, and adherence to optimal medical therapy [10]. At 2-year, the SYNTAX-Ⅱ PCI cohort was superior to the predefined SYNTAX-I PCI cohort and comparable to the predefined SYNTAX-I CABG cohort in terms of a composite of death, any stroke, myocardial infarction, or revascularization [11]. Notably, there was no significant difference in the cumulative 2-year incidences of myocardial infarction and revascularization between SYNTAX-Ⅱ PCI and SYNTAX-I CABG. As opposed to the SYNTAX-Ⅱ study, the excess risk of PCI relative to CABG in the present study was substantial for myocardial infarction and revascularization. The difference might at least partly be explained by the lack of physiologic lesion assessment and adherence to optimal medical therapy, high-intensity statins therapy in particular, in the present study. Moreover, we are not certain whether optimal stent expansion was confirmed by IVUS, although IVUS was used in 86% of patients in the PCI group. Currently, OPTIVUS Complex PCI (Optimal Intravascular Ultrasound Guided Complex Percutaneous Coronary Intervention) study is ongoing, in which we explore the achievement of optimal stent expansion by adhering to the OPTIVUS criteria for the final minimal stent cross-sectional area by IVUS in patients with left main CAD and/or multi-vessel coronary revascularization including LAD. Moreover, in the OPTIVUS Complex PCI study, we strongly recommend physiologic lesion assessment, adoption of trans-radial approach, adherence to optimal medical therapy, and avoidance of scheduled follow-up angiography after PCI to optimize the long-term clinical outcomes, which will be compared historically with those in the present study.

The current study has several limitations. First, this is a retrospective observational study. Therefore, selection bias and unmeasured confounding cannot be excluded despite extensive statistical adjustment, because differences in baseline clinical and procedural characteristics between the groups were substantial. Second, the p-value of all-cause death and non-cardiovascular death was not statistically significant but were borderline. Therefore, we could not deny that the mortality risk might be higher in the PCI group than in the CABG group. Third, there was an increase in any coronary revascularization in the PCI group around 1 year, which might be influenced by routine follow-up coronary angiography. We performed a landmark analysis at 1 year and at 2 years and evaluated outcomes with early events excluded (S2 Fig). Fourth, complete revascularization in the current study was based on anatomical but not physiological or functional evaluation. In comparison with medical therapy only, fractional flow reserve (FFR)-guided PCI with medical therapy was associated with decreased major adverse cardiovascular events in patients with stable CAD. Moreover, patients without hemodynamically significant stenoses had a favorable long-term outcome with medical therapy alone [12]. Therefore, the very low rate of evaluation by FFR before treatment in the current analysis is likely to have affected clinical outcomes in the PCI group, requiring much caution to interpret the results of the present study. Finally, the clinical practices in the present study conducted nearly 10 years ago would certainly have been different from the contemporary practice.

In conclusion, in this observational study, PCI with new-generation DES as compared with CABG was associated with excess long-term risk for major cardiovascular events in patients who underwent multi-vessel coronary revascularization including LAD.

## Supporting information

S1 Appendix List of participating centers and investigators for the CREDO-Kyoto PCI/CABG registry Cohort-3. (DOCX)

S2 Appendix List of clinical research coordinators. (DOCX)

S3 Appendix List of clinical event committee members. (DOCX)

S1 Method Propensity score matching analysis. (DOCX)

S2 Method Landmark analysis at 1 year and at 2 years for any coronary revascularization. (DOCX)

S1 Fig Kaplan-Meier event curves for clinical outcomes in patients with two-vessel disease including LAD. (TIF)

S2 Fig Kaplan-Meier event curves for clinical outcomes in patients with two-vessel or three-vessel disease including proximal LAD. (TIF)

S3 Fig Landmark analysis at (A)1 year and (B)2 years in any coronary revascularization.
(TIF)

S4 Fig Kaplan-Meier event curves for target-vessel revascularization and non-target vessel revascularization in patients with complete revascularization and those with incomplete revascularization. (TIF)

S1 Table Clinical outcomes: PCI group versus CABG group in patients with two-vessel disease who underwent multi-vessel revascularization including LAD. (DOCX)

S2 Table Clinical outcomes: PCI group versus CABG group in patients with two-vessel or three-vessel disease who underwent multi-vessel revascularization including proximal LAD. (DOCX)

S3 Table Baseline characteristics, medications, angiographic and procedural characteristics during the index hospitalization in the propensity score-matched cohort. (DOCX)

S4 Table Clinical outcomes in the propensity score-matched cohort. (DOCX)

## Abbreviations

CABG: coronary arterial bypass grafting
CI: confidence interval
CTO: chronic total occlusion
HR: hazard ratio
MI: myocardial infarction
MVD: multi-vessel disease
PCI: percutaneous coronary intervention
RCT: randomized controlled trial
