# Supplementary material for: Percutaneous coronary intervention using new-generation drug-eluting stents versus coronary arterial bypass grafting in stable patients with multi-vessel coronary artery disease: From the CREDO-Kyoto PCI/CABG registry Cohort-3
Source: PLoS One. 2022 Sep 29;17(9):e0267906. doi: 10.1371/journal.pone.0267906 (PMC9521921; doi:10.1371/journal.pone.0267906)
Supplement: S2 Appendix — (DOCX) [file pone.0267906.s002.docx]

**S2 Appendix. List of Clinical Research Coordinators.**

Research Institute for Production Development:

Sakiko Arimura, Yumika Fujino, Miya Hanazawa, Chikako Hibi, Risa Kato, Yui Kinoshita, Kumiko Kitagawa, Masayo Kitamura, Takahiro Kuwahara, Satoko Nishida, Naoko Okamoto, Yuki Sato, Saori Tezuka, Marina Tsuda, Miyuki Tsumori, Misato Yamauchi, Itsuki Yamazaki
