# Supplementary material for: Percutaneous coronary intervention using new-generation drug-eluting stents versus coronary arterial bypass grafting in stable patients with multi-vessel coronary artery disease: From the CREDO-Kyoto PCI/CABG registry Cohort-3
Source: PLoS One. 2022 Sep 29;17(9):e0267906. doi: 10.1371/journal.pone.0267906 (PMC9521921; doi:10.1371/journal.pone.0267906)
Supplement: S3 Appendix — (DOCX) [file pone.0267906.s003.docx]

**S3 Appendix. List of Clinical Event Committee Members.**

Masayuki Fuki (Kyoto University Hospital), Eri Kato (Kyoto University Hospital),

Yukiko Matsumura-Nakano (Kyoto University Hospital), Kenji Nakatsuma (Mitsubishi Kyoto Hospital), Hiroki Shiomi (Kyoto University Hospital), Yasuaki Takeji (Kyoto University Hospital), Hidenori Yaku (Mitsubishi Kyoto Hospital), Erika Yamamoto (Kyoto University Hospital), Ko Yamamoto (Kyoto University Hospital), Yugo Yamashita (Kyoto University Hospital), Yusuke Yoshikawa (Kyoto University Hospital), Hiroki Watanabe (Japanese Red Cross Wakayama Medical Center)
