# Supplementary material for: Percutaneous coronary intervention using new-generation drug-eluting stents versus coronary arterial bypass grafting in stable patients with multi-vessel coronary artery disease: From the CREDO-Kyoto PCI/CABG registry Cohort-3
Source: PLoS One. 2022 Sep 29;17(9):e0267906. doi: 10.1371/journal.pone.0267906 (PMC9521921; doi:10.1371/journal.pone.0267906)
Supplement: S1 Method — (DOCX) [file pone.0267906.s004.docx]

**S1 Method. Propensity Score Matching Analysis.**

We computed the propensity score by using logistic regression analysis with 14 independent variables potentially influencing the choice of mode of coronary revascularization (age ≥75 years, diabetes, heart failure, prior stroke, eGFR <30 mL/min/1.73m2 without hemodialysis, hemodialysis, anemia, thrombocytopenia, chronic obstructive pulmonary disease, liver cirrhosis, active malignancy, severe frailty, target of proximal left anterior descending coronary artery, and target of chronic total occlusion). Using only the propensity score, patients in the CABG group were matched to PCI patients using a 1:1 greedy matching strategy. This resulted in 797 patients with CABG matched to 797 patients with PCI. Clinical outcomes were compared between the PCI and CABG groups in the propensity score-matched cohort. Cumulative incidence of the outcome measures was estimated by the Kaplan-Meier method, and the differences were assessed with the log-rank test. The effects of PCI relative to CABG for the outcome measures were expressed as hazard ratios (HRs) and their 95% confidence intervals (CIs). The HRs were estimated by the Cox proportional hazard models.
