# Supplementary material for: Percutaneous coronary intervention using new-generation drug-eluting stents versus coronary arterial bypass grafting in stable patients with multi-vessel coronary artery disease: From the CREDO-Kyoto PCI/CABG registry Cohort-3
Source: PLoS One. 2022 Sep 29;17(9):e0267906. doi: 10.1371/journal.pone.0267906 (PMC9521921; doi:10.1371/journal.pone.0267906)
Supplement: S2 Method — (DOCX) [file pone.0267906.s005.docx]

**S2 Method. Landmark analysis at 1 year and at 2 years for any coronary revascularization.**

We performed 1-year and 2-year landmark analyses to estimate the cumulative incidence of any coronary revascularization within or beyond 1 year or 2 years after the index coronary revascularization. In the analysis within 1 year or 2 years, the underlying proportional hazard assumptions in the Cox model for all the variables including the primary variable (PCI versus CABG) were not confirmed. Therefore, odds ratio and 95% confidence intervals were estimated using logistic regression models. Beyond 1 year or 2 years after the index procedure, we used the Cox proportional models as in the main analysis.
