# Supplementary figures and images for: Percutaneous coronary intervention using new-generation drug-eluting stents versus coronary arterial bypass grafting in stable patients with multi-vessel coronary artery disease: From the CREDO-Kyoto PCI/CABG registry Cohort-3
Source: PLoS One. 2022 Sep 29;17(9):e0267906. doi: 10.1371/journal.pone.0267906 (PMC9521921; doi:10.1371/journal.pone.0267906)

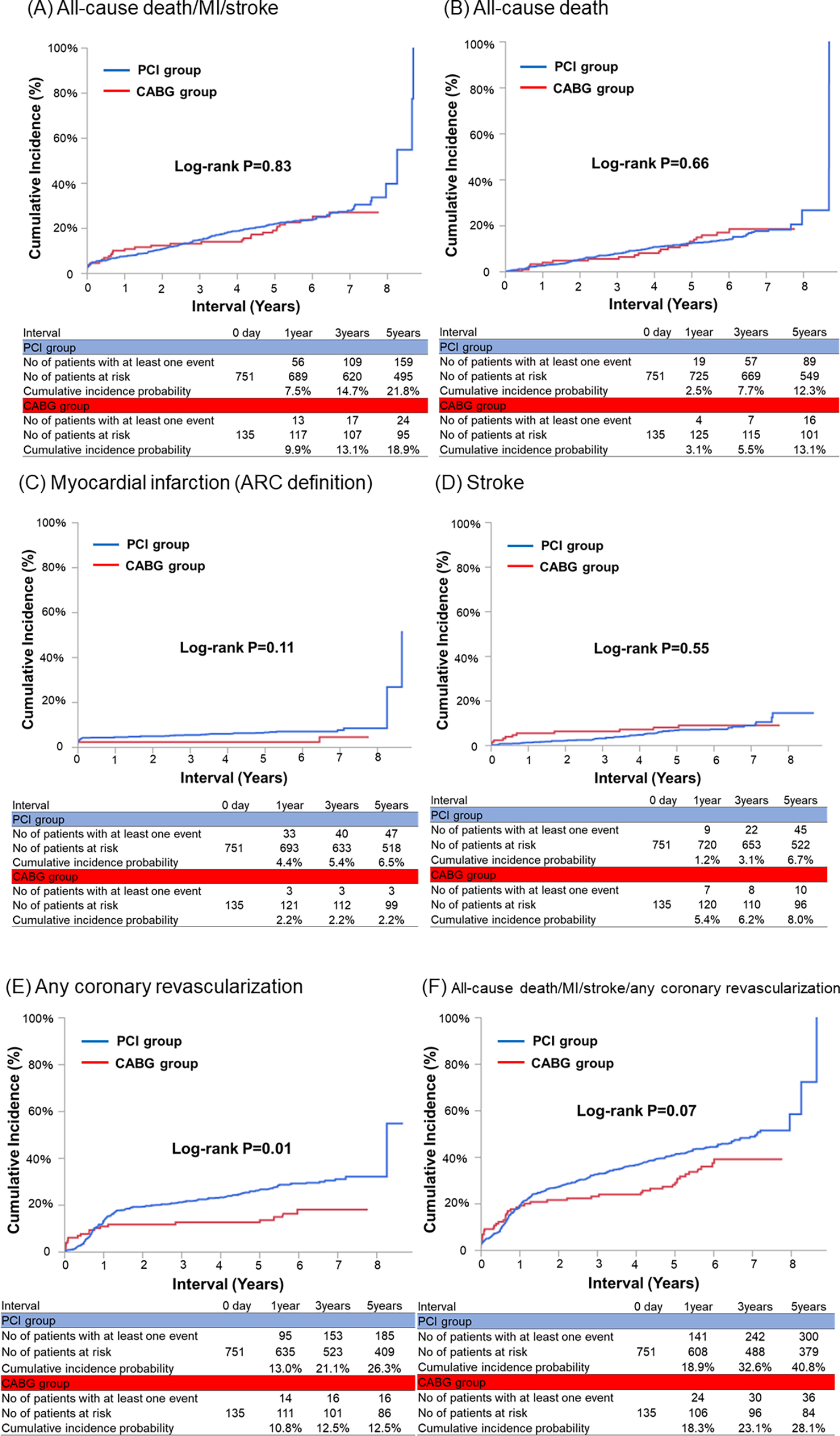

Supplement: S1 Fig — (TIF) [file pone.0267906.s006.TIF]

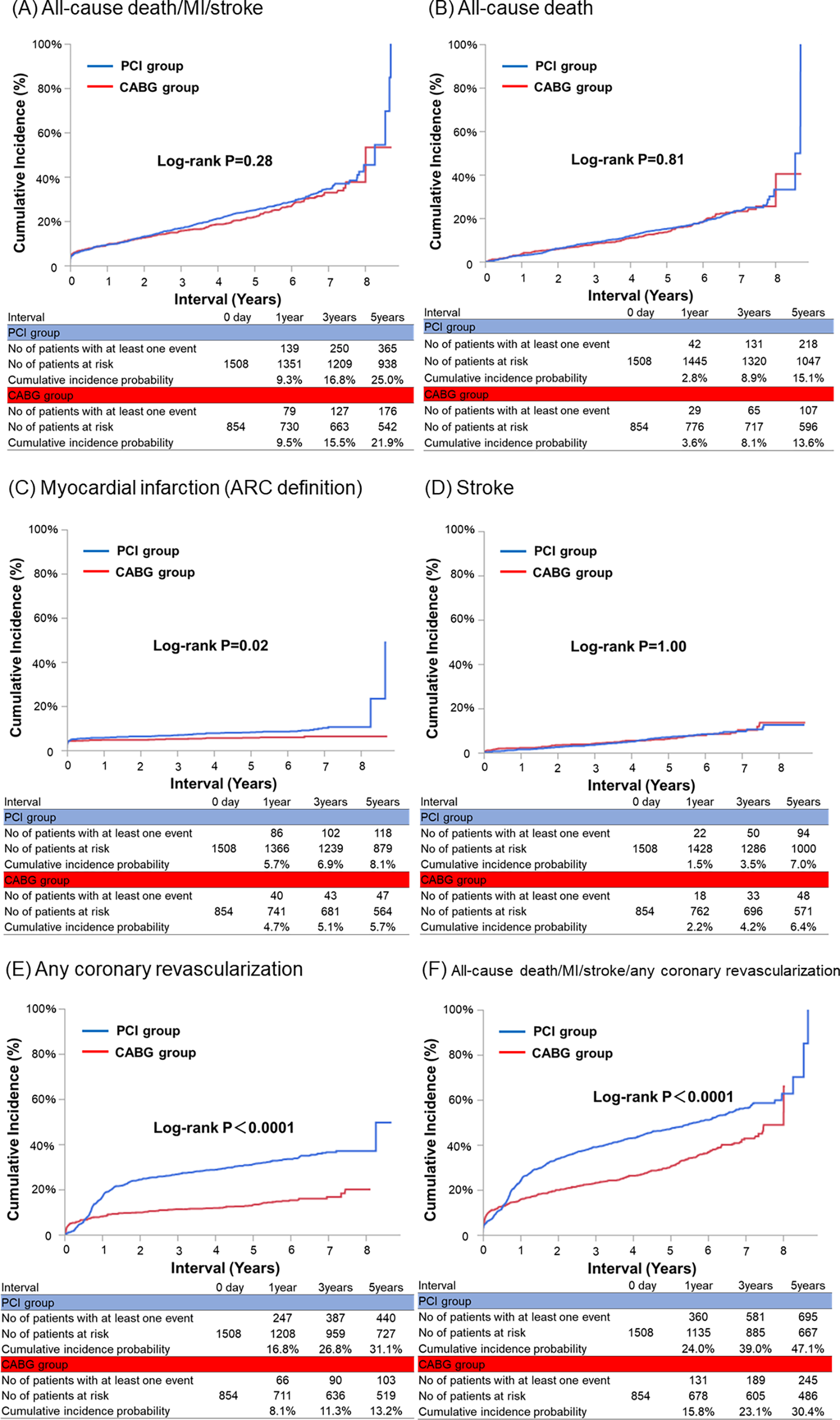

Supplement: S2 Fig — (TIF) [file pone.0267906.s007.TIF]

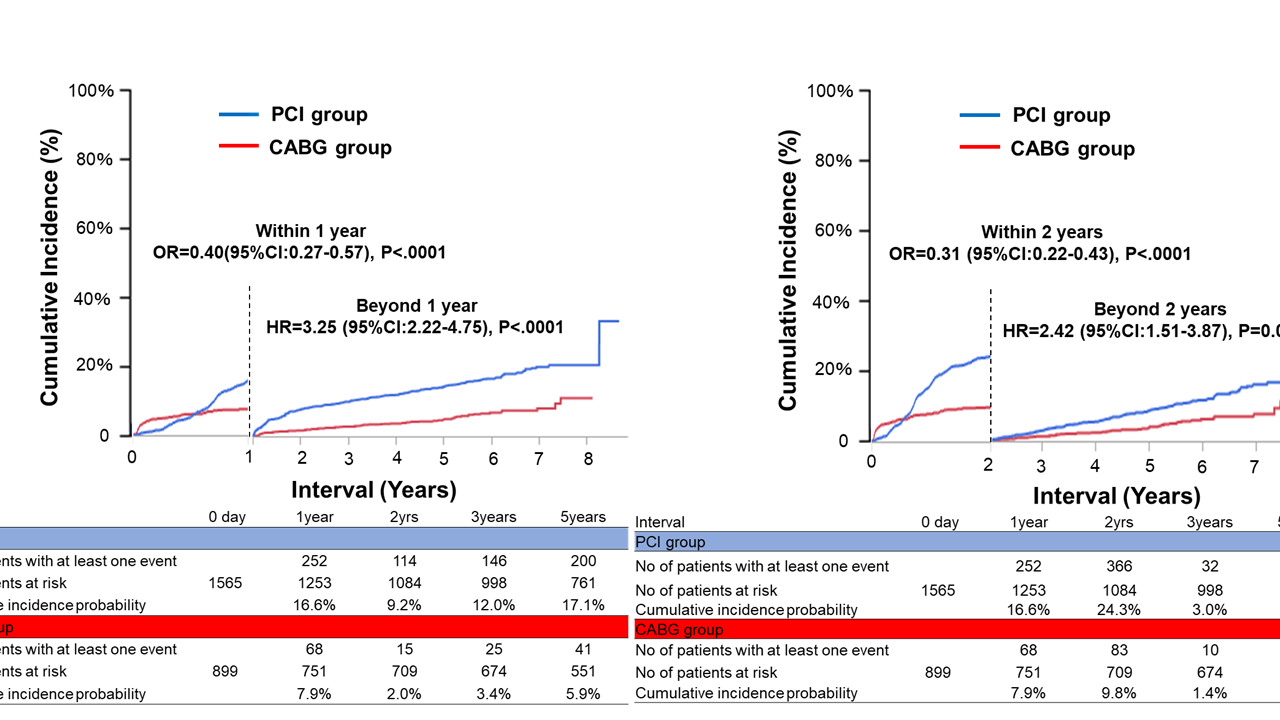

Supplement: S3 Fig — Landmark analysis at (A)1 year and (B)2 years in any coronary revascularization. (TIF) [file pone.0267906.s008.TIF]

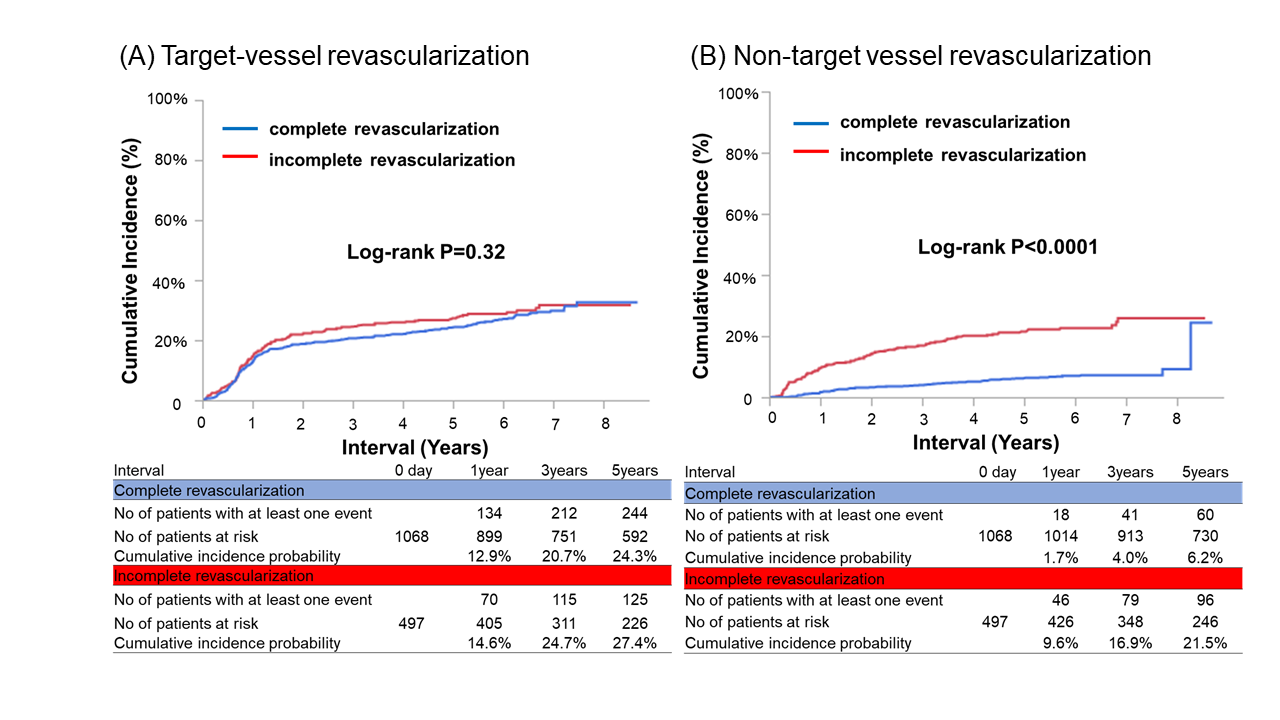

Supplement: S4 Fig — (TIF) [file pone.0267906.s009.TIF]
