# Supplementary material for: Percutaneous coronary intervention using new-generation drug-eluting stents versus coronary arterial bypass grafting in stable patients with multi-vessel coronary artery disease: From the CREDO-Kyoto PCI/CABG registry Cohort-3
Source: PLoS One. 2022 Sep 29;17(9):e0267906. doi: 10.1371/journal.pone.0267906 (PMC9521921; doi:10.1371/journal.pone.0267906)
Supplement: S1 Table — (DOCX) [file pone.0267906.s010.docx]

**S1 Table. Clinical Outcomes: PCI group versus CABG group in patients with two-vessel disease who underwent multi-vessel revascularization including LAD.**

| **Variables** | | | | **PCI group** | **CABG group** | **Crude** | **P value** | **Adjusted** | **P value** |
| --- | --- | --- | --- | --- | --- | --- | --- | --- | --- |
|  |  |  |  | **N of patients with events** | **N of patients with events** | **HR** |  | **HR** |  |
|  |  |  |  | **(Cumulative incidence)** | **(Cumulative incidence)** | **(95％CI)** |  | **(95％CI)** |  |
|  |  |  |  | **N=751** | **N=135** |  |  |  |  |
| **Primary outcome measure** | | | |  |  |  |  |  |  |
|  | **A composite of death, MI, or stroke** | | | 192(21.8%) | 31 (18.9%) | 1.04 | 0.83 | 1.20 | 0.38 |
|  |  |  |  |  |  | (0.72-1.55) |  | (0.80-1.80) |  |
| **Secondary outcome measures** | | | |  |  |  |  |  |  |
|  | **All-cause death** | | | 113 (12.3%) | 21 (13.1%) | 0.90 | 0.66 | 0.91 | 0.73 |
|  |  |  |  |  |  | (0.58-1.47) |  | (0.55-1.51) |  |
|  |  | **Cardiovascular death** | | 62 (6.6%) | 7 (5.7%) | 1.47 | 0.31 | 1.38 | 0.41 |
|  |  |  |  |  |  | (0.72-3.54) |  | (0.66-3.34) |  |
|  |  | **Cardiac death** | | 45 (4.6%) | 5 (4.1%) | 1.50 | 0.37 | 1.38 | 0.48 |
|  |  |  |  |  |  | (0.65-4.32) |  | (0.59-4.04) |  |
|  |  |  | **Sudden cardiac death** | 12 (1.5%) | 2 (1.7%) | 1.04 | 0.96 | NA | NA |
|  |  |  |  |  |  | (0.28-6.67) |  | NA |  |
|  |  | **Non-cardiovascular death** | | 51 (6.1%) | 14 (7.8%) | 0.61 | 0.12 | 0.66 | 0.17 |
|  |  |  |  |  |  | (0.35-1.15) |  | (0.36-1.20) |  |
|  |  | **Non-cardiac death** | | 68 (8.0%) | 16 (9.4%) | 0.71 | 0.24 | 0.75 | 0.30 |
|  |  |  |  |  |  | (0.42-1.27) |  | (0.43-1.30) |  |
|  | **Myocardial infarction** | | |  |  |  |  |  |  |
|  |  | **ARC definition** | | 53 (6.5%) | 4 (2.2%) | 2.25 | 0.08 | 2.43 | 0.09 |
|  |  |  |  |  |  | (0.92-7.45) |  | (0.87-6.80) |  |
|  |  |  | **Periprocedural MI** | 33 (4.2%) | 3 (2.2%) | 1,93 | 0.23 | 2.01 | 0.25 |
|  |  |  |  |  |  | (0.69-8.01) |  | (0.61-6.64) |  |
|  |  |  | **Spontaneous MI** | 20 (2.4%) | 1 (0%) | 3.17 | 0.18 | NA | NA |
|  |  |  |  |  |  | (0.66-57.0) |  | NA |  |
|  |  | **ARTS definition** | | 43 (5.2%) | 2 (0.7%) | 3.61 | 0.03 | 4.06 | 0.05 |
|  |  |  |  |  |  | (1.11-22.1) |  | (0.97-16.9) |  |
|  | **Definite stent thrombosis or symptomatic graft occlusion** | | | 6 (0.9%) | 1(0.7%) | 1.05 | 0.96 | NA | NA |
|  |  |  |  |  |  | (0.18-19.9) |  | NA |  |
|  | **Stroke** | | | 55 (6.7%) | 11 (8.0%) | 0.82 | 0.56 | 0.87 | 0.68 |
|  |  |  |  |  |  | (0.45-1.66) |  | (0.45-1.67) |  |
|  |  | **Ischemic stroke** | | 41 (4.9%) | 9 (6.4%) | 0.75 | 0.44 | 0.81 | 0.57 |
|  |  |  |  |  |  | (0.38-1.64) |  | (0.39-1.68) |  |
|  |  | **Hemorrhagic stroke** | | 16 (2.0%) | 3 (2.4%) | 0.90 | 0.87 | NA | NA |
|  |  |  |  |  |  | (0.30-3.88) |  | NA |  |
|  |  | **Major stroke** | | 39 (4.8%) | 10 (7.2%) | 0.63 | 0.22 | 0.64 | 0.24 |
|  |  |  |  |  |  | (0.33-1.34) |  | (0.33-1.37) |  |
|  | **Hospitalization for HF** | | | 63 (8.0%) | 13 (8.1%) | 0.80 | 0.47 | 0.98 | 0.94 |
|  |  |  |  |  |  | (0.46-1.52) |  | (0.53-1.80) |  |
|  | **Major bleeding** | | |  |  |  |  |  |  |
|  |  | **BARC type 3,4, or 5** | | 101 (12.4%) | 47 (34.0%) | 0.31 | <.0001 | 0.33 | <.0001 |
|  |  |  |  |  |  | (0.22-0.44) |  | (0.22-0.48) |  |
|  |  |  | **In-hospital bleeding** | 13 (1.7%) | 34(25.2%) | 0.07 | <.0001 | 0.06 | <.0001 |
|  |  |  |  |  |  | (0.03-0.12) |  | (0.03-0.12) |  |
|  |  |  | **Out-of-hospital bleeding** | 88 (10.7%) | 13 (8.9%) | 1.17 | 0.59 | 1.62 | 0.13 |
|  |  |  |  |  |  | (0.68-2.19) |  | (0.87-3.03) |  |
|  |  | **BARC type 3** | | 90 (11.0%) | 20 (13.8%) | 0.76 | 0.29 | 0.96 | 0.87 |
|  |  |  |  |  |  | (0.48-1.28) |  | (0.57-1.62) |  |
|  |  | **BARC type 4** | | 5 (0.7%) | 26 (19.3%) | 0.03 | <.0001 | 0.03 | <.0001 |
|  |  |  |  |  |  | (0.01-0.08) |  | (0.01-0.09) |  |
|  |  | **BARC type 5** | | 6 (0.7%) | 1 (0.8%) | 1.02 | 0.98 | NA | NA |
|  |  |  |  |  |  | (0.17-19.3) |  | NA |  |
|  |  | **GUSTO moderate or severe** | | 85 (10.3%) | 80 (59.5%) | 0.12 | <.0001 | 0.11 | <.0001 |
|  |  |  |  |  |  | (0.09-0.17) |  | (0.08-0.16) |  |
|  |  |  | **In-hospital bleeding** | 6 (0.8%) | 75 (55.6%) | 0.01 | <.0001 | 0.01 | <.0001 |
|  |  |  |  |  |  | (0.005-0.03) |  | (0.006-0.03) |  |
|  |  |  | **Out-of-hospital bleeding** | 79 (9.5%) | 5 (4.0%) | 2.75 | 0.01 | 3.41 | 0.009 |
|  |  |  |  |  |  | (1.23-7.82) |  | (1.37-8.50) |  |
|  |  | **GUSTO severe** | | 50 (6.1%) | 21 (14.6%) | 0.38 | 0.0006 | 0.36 | 0.0002 |
|  |  |  |  |  |  | (0.23-0.65) |  | (0.21-0.61) |  |
|  | **Target-vessel revascularization** | | | 174 (21.7%) | 17 (11.7%) | 1.77 | 0.02 | 1.71 | 0.04 |
|  |  |  |  |  |  | (1.11-3.02) |  | (1.02-2.89) |  |
|  |  | **Ischemia-driven target-vessel revascularization** | | 85 (9.8%) | 12 (7.9%) | 1.18 | 0.59 | 1.11 | 0.73 |
|  |  |  |  |  |  | (0.67-2.27) |  | (0.63-2.16) |  |
|  | **Any coronary revascularization** | | | 204 (26.3%) | 20 (12.5%) | 1.80 | 0.006 | 1.61 | 0.04 |
|  |  |  |  |  |  | (1.17-2.95) |  | (1.02-2.68) |  |
|  |  | **Ischemia-driven any coronary revascularization** | | 101 (12.4%) | 15 (8.7%) | 1.12 | 0.68 | 1.03 | 0.93 |
|  |  |  |  |  |  | (0.67-2.00) |  | (0.58-1.82) |  |
|  | **A composite of death, MI, stroke, or any coronary revascularization** | | | 339 (40.8%) | 46 (28.1%) | 1.33 | 0.06 | 1.34 | 0.08 |
|  |  |  |  |  |  | (0.99-1.83) |  | (0.97-1.86) |  |
