# Supplementary material for: Percutaneous coronary intervention using new-generation drug-eluting stents versus coronary arterial bypass grafting in stable patients with multi-vessel coronary artery disease: From the CREDO-Kyoto PCI/CABG registry Cohort-3
Source: PLoS One. 2022 Sep 29;17(9):e0267906. doi: 10.1371/journal.pone.0267906 (PMC9521921; doi:10.1371/journal.pone.0267906)
Supplement: S2 Table — (DOCX) [file pone.0267906.s011.docx]

**S2 Table. Clinical Outcomes: PCI group versus CABG group in patients with two-vessel or three-vessel disease who underwent multi-vessel revascularization including proximal LAD.**

| **Variables** | | | | **PCI group** | **CABG group** | **Crude** | **P value** | **Adjusted** | **P value** |
| --- | --- | --- | --- | --- | --- | --- | --- | --- | --- |
|  |  |  |  | **N of patients with events** | **N of patients with events** | **HR** |  | **HR** |  |
|  |  |  |  | **(Cumulative incidence)** | **(Cumulative incidence)** | **(95％CI)** |  | **(95％CI)** |  |
|  |  |  |  | **N=1508** | **N=854** |  |  |  |  |
| **Primary outcome measure** | | | |  |  |  |  |  |  |
|  | **A composite of death, MI, or stroke** | | | 455(25.0%) | 228 (21.9%) | 1.09 | 0.28 | 1.22 | 0.06 |
|  |  |  |  |  |  | (0.93-1.28) |  | (0.99-1.49) |  |
| **Secondary outcome measures** | | | |  |  |  |  |  |  |
|  | **All-cause death** | | | 291(15.1%) | 153(13.6%) | 1.02 | 0.80 | 1.14 | 0.31 |
|  |  |  |  |  |  | (0.84-1.25) |  | (0.89-1.45) |  |
|  |  | **Cardiovascular death** | | 142(7.4%) | 89(8.7%) | 0.86 | 0.27 | 1.02 | 0.90 |
|  |  |  |  |  |  | (0.66-1.13) |  | (0.73-1.43) |  |
|  |  | **Cardiac death** | | 102(5.3%) | 63(6.7%) | 0.87 | 0.41 | 1.04 | 0.84 |
|  |  |  |  |  |  | (0.64-1.20) |  | (0.70-1.54) |  |
|  |  |  | **Sudden cardiac death** | 27(1.7%) | 19(2.1%) | 0.77 | 0.39 | 0.92 | 0.80 |
|  |  |  |  |  |  | (0.43-1.41) |  | (0.51-1.68) |  |
|  |  | **Non-cardiovascular death** | | 149(8.3%) | 64(5.4%) | 1.25 | 0.13 | 1.29 | 0.17 |
|  |  |  |  |  |  | (0.94-1.69) |  | (0.89-1.87) |  |
|  |  | **Non-cardiac death** | | 189(10.3%) | 90(7.5%) | 1.13 | 0.34 | 1.22 | 0.22 |
|  |  |  |  |  |  | (0.88-1.46) |  | (0.89-1.68) |  |
|  | **Myocardial infarction** | | |  |  |  |  |  |  |
|  |  | **ARC definition** | | 131(8.1%) | 49(5.7%%) | 1.47 | 0.02 | 1.65 | 0.02 |
|  |  |  |  |  |  | (1.07-2.07) |  | (1.08-2.51) |  |
|  |  |  | **Periprocedural MI** | 83(5.4%) | 35(4.1%) | 1.33 | 0.15 | 1.54 | 0.10 |
|  |  |  |  |  |  | (0.90-1.99) |  | (0.92-2.59) |  |
|  |  |  | **Spontaneous MI** | 48(2.7%) | 14(1.6%) | 1.82 | 0.049 | 2.19 | 0.01 |
|  |  |  |  |  |  | (1.00-3.30) |  | (1.19-4.01) |  |
|  |  | **ARTS definition** | | 91(5.5%) | 24(2.8%) | 2.08 | 0.0007 | 2.45 | 0.002 |
|  |  |  |  |  |  | (1.35-3.33) |  | (1.39-4.31) |  |
|  | **Definite stent thrombosis or symptomatic graft occlusion** | | | 9(0.7%) | 8(1.1%) | 0.62 | 0.32 | NA | NA |
|  |  |  |  |  |  | (0.24-1.64) |  | NA |  |
|  | **Stroke** | | | 116(7.0%) | 63(6.4%) | 1.00 | 1.00 | 1.17 | 0.44 |
|  |  |  |  |  |  | (0.74-1.37) |  | (0.79-1.74) |  |
|  |  | **Ischemic stroke** | | 92(5.4%) | 50(5.0%) | 1.00 | 1.00 | 1.17 | 0.50 |
|  |  |  |  |  |  | (0.71-1.42) |  | (0.75-1.83) |  |
|  |  | **Hemorrhagic stroke** | | 31(2.0%) | 16(1.7%) | 1.06 | 0.85 | 1.09 | 0.79 |
|  |  |  |  |  |  | (0.59-1.98) |  | (0.59-2.01) |  |
|  |  | **Major stroke** | | 86(5.3%) | 48(5.0%) | 0.98 | 0.90 | 1.17 | 0.51 |
|  |  |  |  |  |  | (0.69-1.40) |  | (0.74-1.84) |  |
|  | **Hospitalization for HF** | | | 150(9.4%) | 91(10.0%) | 0.87 | 0.30 | 1.02 | 0.92 |
|  |  |  |  |  |  | (0.67-1.13) |  | (0.73-1.42) |  |
|  | **Major bleeding** | | |  |  |  |  |  |  |
|  |  | **BARC type 3,4, or 5** | | 235(15.1%) | 326(37.0%) | 0.34 | <.0001 | 0.36 | <.0001 |
|  |  |  |  |  |  | (0.29-0.40) |  | (0.29-0.45) |  |
|  |  |  | **In-hospital bleeding** | 38(2.5%) | 259(30.3) | 0.08 | <.0001 | 0.09 | <.0001 |
|  |  |  |  |  |  | (0.06-0.11) |  | (0.06-0.13) |  |
|  |  |  | **Out-of-hospital bleeding** | 197(12.6%) | 67(6.8%) | 1.63 | 0.00 | 1.83 | 0.0007 |
|  |  |  |  |  |  | (1.23-2.15) |  | (1.29-2.60) |  |
|  |  | **BARC type 3** | | 211(13.7%) | 122(13.5%) | 0.94 | 0.60 | 1.02 | 0.88 |
|  |  |  |  |  |  | (0.75-1.18) |  | (0.77-1.36) |  |
|  |  | **BARC type 4** | | 12(0.7%) | 194(22.6%) | 0.03 | <.0001 | 0.04 | <.0001 |
|  |  |  |  |  |  | (0.02-0.06) |  | (0.02-0.08) |  |
|  |  | **BARC type 5** | | 12(0.8%) | 10(1.2%) | 0.65 | 0.32 | NA | NA |
|  |  |  |  |  |  | (0.28-1.54) |  | NA |  |
|  |  | **GUSTO moderate or severe** | | 195(12.5%) | 536(62.2%) | 0.15 | <.0001 | 0.16 | <.0001 |
|  |  |  |  |  |  | (0.13-0.18) |  | (0.13-0.20) |  |
|  |  |  | **In-hospital bleeding** | 22(1.5%) | 515(60.3%) | 0.02 | <.0001 | 0.02 | <.0001 |
|  |  |  |  |  |  | (0.01-0.03) |  | (0.02-0.04) |  |
|  |  |  | **Out-of-hospital bleeding** | 173(11.0%) | 21(2.0%) | 4.65 | <.0001 | 6.22 | <.0001 |
|  |  |  |  |  |  | (3.03-7.53) |  | (3.64-10.6) |  |
|  |  | **GUSTO severe** | | 108(6.8%) | 97(11.0%) | 0.59 | 0.0002 | 0.59 | <.0001 |
|  |  |  |  |  |  | (0.45-0.77) |  | (0.42-0.83) |  |
|  | **Target-vessel revascularization** | | | 398(25.6%) | 105(11.9%) | 2.20 | <.0001 | 2.53 | <.0001 |
|  |  |  |  |  |  | (1.78-2.74) |  | (1.92-3.35) |  |
|  |  | **Ischemia-driven target-vessel revascularization** | | 197(11.9%) | 65(7.1%) | 1.68 | 0.0003 | 1.60 | <.0001 |
|  |  |  |  |  |  | (1.27-2.23) |  | (1.12-2.28) |  |
|  | **Any coronary revascularization** | | | 477(31.1%) | 118(13.2%) | 2.40 | <.0001 | 2.71 | <.0001 |
|  |  |  |  |  |  | (1.97-2.95) |  | (2.09-3.52) |  |
|  |  | **Ischemia-driven any coronary revascularization** | | 234(14.6%) | 72(7.6%) | 1.82 | <.0001 | 1.60 | 0.006 |
|  |  |  |  |  |  | (1.40-2.37) |  | (1.15-2.23) |  |
|  | **A composite of death, MI, stroke, or any coronary revascularization** | | | 781(47.1%) | 306(30.4%) | 1.56 | <.0001 | 1.68 | <.0001 |
|  |  |  |  |  |  | (1.37-1.78) |  | (1.41-1.99) |  |
