# Supplementary material for: Percutaneous coronary intervention using new-generation drug-eluting stents versus coronary arterial bypass grafting in stable patients with multi-vessel coronary artery disease: From the CREDO-Kyoto PCI/CABG registry Cohort-3
Source: PLoS One. 2022 Sep 29;17(9):e0267906. doi: 10.1371/journal.pone.0267906 (PMC9521921; doi:10.1371/journal.pone.0267906)
Supplement: S3 Table — (DOCX) [file pone.0267906.s012.docx]

**S3 Table. Baseline Characteristics, Medications, angiographic and procedural characteristics during the Index Hospitalization in the Propensity Score-Matched Cohort.**

| Variables | PCI group | CABG group | P value |
| --- | --- | --- | --- |
|  | N=797 | N=797 |  |
| **Clinical characteristics** |  |  |  |
| Age | 68.7±10.4 | 68.6±9.7 | 0.88 |
| >=75years | 248(31%) | 242(30％) | 0.74 |
| Male | 589(74％) | 618(78％) | 0.09 |
| Body mass index (kg/m²) | 24.2±3.8 (N=793) | 23.8±3.4 (N=796) | 0.02 |
| <25.0kg/m² | 498(62％) | 526(66％) | 0.14 |
| Hypertension | 687(86%) | 684(86%) | 0.83 |
| Diabetes mellitus | 418(52%) | 418(52%) | 1.00 |
| requiring insulin therapy | 100(13%) | 166(21%) | <0.0001 |
| Current smoking | 181(23%) | 142(18%) | 0.02 |
| Heart failure | 198(25%) | 189(24%) | 0.60 |
| History of heart failure | 72(9.0%) | 136(17%) | <0.0001 |
| Current heart failure | 153(19%) | 84(11%) | <0.0001 |
| Clinical presentation |  |  | 0.0007 |
| Stable angina | 462(58%) | 508(64%) |  |
| Unstable angina | 6(0.8%%) | 10(1.3%) |  |
| AMI | 31(3.9%) | 15(1.9%) |  |
| Silent myocardial ischemia | 65(8.2%) | 86(11%) |  |
| Old myocardial infarction without angina | 96(12%) | 60(7.5%) |  |
| Coronary stenosis without documentation of myocardial ischemia | 137(17%) | 118(15%) |  |
| LVEF | 57±14 | 59±14 | 0.02 |
| LVEF <= 40% | 94/722(13%) | 90/764(12%) | 0.47 |
| Mitral regurgitation grade>=3/4 | 47/726(6.5%) | 61/764(8.0%) | 0.26 |
| Previous myocardial infarction | 202(25%) | 197(25%) | 0.77 |
| Previous symptomatic stroke | 131(16%) | 128(16%) | 0.84 |
| Peripheral vascular disease | 89(11%) | 111(14%) | 0.10 |
| eGFR<30mL/min/1.73m² or hemodialysis | 96(12%) | 98(12%) | 0.88 |
| eGFR<30mL/min/1.73m² without hemodialysis | 38(4.8%) | 38(4.8%) | 1.00 |
| Hemodialysis | 58(7.3%) | 60(7.5%) | 0.85 |
| Atrial fibrillation | 71(8.9%) | 62(7.8%) | 0.41 |
| Anemia (hemoglobin<11.0g/dL) | 116(15%) | 125(16%) | 0.53 |
| Thrombocytopenia (Platelet < 100*10⁹/L) | 16(2.0%) | 19(2.4%) | 0.61 |
| Chronic obstructive pulmonary disease | 31(3.9%) | 32(4.0%) | 0.90 |
| Liver cirrhosis | 22(2.8%) | 24(3.0%) | 0.76 |
| Malignancy | 87(11%) | 85(11%) | 0.87 |
| Active malignancy | 8(1.0%) | 12(1.5%) | 0.37 |
| Severe frailty | 14(1.8%) | 14(1.8%) | 1.00 |
| **Baseline medications** |  |  |  |
| Antiplatelet therapy |  |  |  |
| Thienopyridine | 795(100%) | 166(21%) | <0.0001 |
| Ticlopidine | 14(1.8%) | 12(7.2%) |  |
| Clopidogrel | 779(98%) | 154(93%) |  |
| Unknown | 2(0.3%) | 0 |  |
| Aspirin | 796(100%) | 784(98%) | 0.0005 |
| Cilostazole | 19(2.4%) | 25(3.1%) | 0.36 |
| Other medications |  |  |  |
| Statin | 614(77%) | 521(65%) | <0.0001 |
| High-intensity statin | 11(1.4%) | 4(0.5%) | 0.06 |
| ACE-I/ARB | 512(64%) | 253(32%) | <0.0001 |
| β blocker | 299(38%) | 449(56%) | <0.0001 |
| Nitrate | 189(24%) | 95(12%) | <0.0001 |
| Calcium channel blocker | 386(48%) | 309(39%) | <0.0001 |
| Nicorandil | 112(14%) | 290(36%) | <0.0001 |
| Oral anticoagulants | 78(9.8%) | 431(54%) | <0.0001 |
| Warfarin | 69(8.7%) | 426(53%) | <0.0001 |
| DOAC | 9(1.1%) | 5(0.6%) | 0.28 |
| Proton pump inhibitor or histamine type-2 receptor blocker | 585(73%) | 736(92%) | <0.0001 |
| Proton pump inhibitor | 498(62%) | 670(84%) | <0.0001 |
| Histamine type-2 receptor blocker | 89(11%) | 68(8.5%) | 0.08 |
| **Angiographic and procedural characteristics** |  |  |  |
| Three-vessel disease | 435(55%) | 676(85%) | <0.0001 |
| Chronic total occlusion (target or non-target) | 400 (50%) | 366 (46%) | 0.09 |
| Location of CTO (target and non-target) |  |  |  |
| LAD | 164(21%) | 153(19%) | 0.49 |
| LCX | 129(16%) | 111(14%) | 0.21 |
| RCA | 189(24%) | 214(27%) | 0.15 |
| Number of target lesion or anastomoses | 2.5±0.7 | 3.4±0.9 | <0.0001 |
| SYNTAX score | 26±8.9(N=417) | 29±7.9(N=535) | <0.0001 |
| Low (<23) | 155/417(37%) | 115/535(22%) | <0.0001 |
| Intermediate (23-32) | 179/417(43%) | 244/535(46%) | 0.41 |
| High (>=33) | 83/417(20%) | 176/535(33%) | <0.0001 |
| Target vessel or anastomoses |  |  |  |
| Proximal LAD | 758(95%) | 761(95%) | 0.72 |
| LCX | 454(57%) | 678(85%) | <0.0001 |
| RCA | 519(65%) | 671(84%) | <0.0001 |
| Target CTO vessel | 347(44%) | 343(43%) | 0.84 |
| Total number of stents | 3(2-4) | - |  |
| Total stent length (mm) | 74(51-102) | - |  |
| Type of DES |  |  |  |
| Everolimus-eluting stent (XIENCE™) use | 483(61%) | - | - |
| Everolimus-eluting stent (PROMUS™) use | 234(29%) | - | - |
| Biolimus-eluting stent (NOBORI™) use | 284(36%) | - | - |
| Zotarolimus-eluting stent (RESOLUTE™) use | 69(8.7%) | - | - |
| Zotarolimus-eluting stent (ENDEAVOR™) use | 9(1.1%) | - | - |
| IVUS or OCT use | 692(87%) | - | - |
| IVUS use | 691(87%) | - | - |
| Number of CTO vessels (target and non-target) |  |  | 0.46 |
|  | 0.6±0.7 | 0.6±0.7 |  |
|  | 1(0-1) | 0(0-1) |  |
| Number of CTO target vessels |  |  | 0.30 |
|  | 0.5±0.6 | 0.5±0.7 |  |
|  | 0(0-1) | 0(0-1) |  |
| Location of target CTO vessels |  |  |  |
| LAD | 150(19%) | 153(19%) | 0.85 |
| LCX | 94(12%) | 97(12%) | 0.82 |
| RCA | 160(20%) | 188(24%) | 0.09 |
| Successful CTO PCI | 302/347 (87%) | - |  |
| Bifurcated lesion | 486(61%) | - |  |
| Side-branch stenting | 81(10%) | - |  |
| Staged PCI | 518(65%) | - |  |
| Complete revascularization | 538(68%) | 673(84%) | <0.0001 |
| Two-vessel disease | 362/362(100%) | 121/121(100%) | - |
| Three-vessel disease | 176/435(40%) | 552/676(82%) | <0.0001 |
| Internal thoracic artery graft use | - | 782(98%) |  |
| Off-pump surgery | - | 462(58%) |  |
