# Supplementary material for: Percutaneous coronary intervention using new-generation drug-eluting stents versus coronary arterial bypass grafting in stable patients with multi-vessel coronary artery disease: From the CREDO-Kyoto PCI/CABG registry Cohort-3
Source: PLoS One. 2022 Sep 29;17(9):e0267906. doi: 10.1371/journal.pone.0267906 (PMC9521921; doi:10.1371/journal.pone.0267906)
Supplement: S4 Table — (DOCX) [file pone.0267906.s013.docx]

**S4 Table. Clinical Outcomes in the Propensity Score-Matched Cohort**

| **Variables** | | | | **PCI group** | **CABG group** | **Crude** | **P value** |
| --- | --- | --- | --- | --- | --- | --- | --- |
|  |  |  |  | **N of patients with events** | **N of patients with events** | **HR** |  |
|  |  |  |  | **(Cumulative incidence)** | **(Cumulative incidence)** | **(95％CI)** |  |
|  |  |  |  | **N=797** | **N=797** |  |  |
| **Primary outcome measure** | | | |  |  |  |  |
|  | **A composite of death, MI, or stroke** | | | 258 (26.4%) | 204 (21.1%) | 1.24 | 0.02 |
|  |  |  |  |  |  | (1.03-1.49) |  |
| **Secondary outcome measures** | | | |  |  |  |  |
|  | **All-cause death** | | | 163 (16.2%) | 134 (12.8%) | 1.17 | 0.85 |
|  |  |  |  |  |  | (0.93-1.47) |  |
|  |  | **Cardiovascular death** | | 79 (8.0%) | 78 (8.2%) | 0.98 | 0.89 |
|  |  |  |  |  |  | (0.72-1.34) |  |
|  |  | **Cardiac death** | | 55 (5.7%) | 55 (6.3%) | 0.97 | 0.86 |
|  |  |  |  |  |  | (0.66-1.41) |  |
|  |  |  | **Sudden cardiac death** | 17 (1.5%) | 15 (2.2%) | 0.85 | 0.66 |
|  |  |  |  |  |  | (0.42-1.71) |  |
|  |  | **Non-cardiovascular death** | | 84 (8.9%) | 56 (5.0%) | 1.44 | 0.03 |
|  |  |  |  |  |  | (1.03-2.03) |  |
|  |  | **Non-cardiac death** | | 108 (11.1%) | 79 (6.9%) | 1.31 | 0.07 |
|  |  |  |  |  |  | (0.98-1.76) |  |
|  | **Myocardial infarction** | | |  |  |  |  |
|  |  | **ARC definition** | | 73 (8.4%) | 43 (5.4%) | 1.68 | 0.006 |
|  |  |  |  |  |  | (1.16-2.47) |  |
|  |  |  | **Periprocedural MI** | 47(5.7%) | 28 (3.5%) | 1.67 | 0.03 |
|  |  |  |  |  |  | (1.05-2.69) |  |
|  |  |  | **Spontaneous MI** | 26 (2.7%) | 15 (1.9%) | 1.68 | 0.11 |
|  |  |  |  |  |  | (0.90-3.24) |  |
|  |  | **ARTS definition** | | 50 (5.5%) | 22 (2.8%) | 2.24 | 0.002 |
|  |  |  |  |  |  | (1.36-3.70) |  |
|  | **Definite stent thrombosis or symptomatic graft occlusion** | | | 5 (0.7%) | 9 (1.3%) | 0.54 | 0.26 |
|  |  |  |  |  |  | (0.17-1.56) |  |
|  | **Stroke** | | | 68 (7.6%) | 60 (6.7%) | 1.10 | 0.59 |
|  |  |  |  |  |  | (0.78-1.56) |  |
|  |  | **Ischemic stroke** | | 53 (5.7%) | 47 (5.2%) | 1.09 | 0.66 |
|  |  |  |  |  |  | (0.74-1.62) |  |
|  |  | **Hemorrhagic stroke** | | 18 (2.2%) | 15 (1.8%) | 1.18 | 0.64 |
|  |  |  |  |  |  | (0.59-2.36) |  |
|  |  | **Major stroke** | | 47 (5.5%) | 44 (5.3%) | 1.04 | 0.84 |
|  |  |  |  |  |  | (0.69-1.58) |  |
|  | **Hospitalization for HF** | | | 89 (10.6%) | 87 (10.1%) | 0.98 | 0.87 |
|  |  |  |  |  |  | (0.73-1.31) |  |
|  | **Major bleeding** | | |  |  |  |  |
|  |  | **BARC type 3,4, or 5** | | 126 (14.7%) | 288 (35.2%) | 0.38 | <.0.0001 |
|  |  |  |  |  |  | (0.31-0.47) |  |
|  |  |  | **In-hospital bleeding** | 20 (2.5%) | 227 (28.5%) | 0.09 | <.0.0001 |
|  |  |  |  |  |  | (0.05-0.13) |  |
|  |  |  | **Out-of-hospital bleeding** | 106 (12.3%) | 61 (6.9%) | 1.70 | 0.0008 |
|  |  |  |  |  |  | (1.24-2.34) |  |
|  |  | **BARC type 3** | | 111 (13.3%) | 104 (12.4%) | 1.03 | 0.82 |
|  |  |  |  |  |  | (0.79-1.35) |  |
|  |  | **BARC type 4** | | 7 (0.7%) | 174 (21.7%) | 0.04 | <.0.0001 |
|  |  |  |  |  |  | (0.02-0.08) |  |
|  |  | **BARC type 5** | | 8 (0.8%) | 10 (1.3%) | 0.77 | 0.59 |
|  |  |  |  |  |  | (0.30-1.96) |  |
|  |  | **GUSTO moderate or severe** | | 99 (11.4%) | 485 (60.3%) | 0.16 | <.0.0001 |
|  |  |  |  |  |  | (0.13-0.20) |  |
|  |  |  | **In-hospital bleeding** | 10 (1.3%) | 464 (58.2%) | 0.02 | <.0.0001 |
|  |  |  |  |  |  | (0.01-0.04) |  |
|  |  |  | **Out-of-hospital bleeding** | 89 (10.1%) | 21 (2.1%) | 4.20 | <.0.0001 |
|  |  |  |  |  |  | (2.67-6.94) |  |
|  |  | **GUSTO severe** | | 54 (6.1%) | 87 (10.7%) | 0.58 | 0.002 |
|  |  |  |  |  |  | (0.41-0.82) |  |
|  | **Target-vessel revascularization** | | | 217 (26.0%) | 102 (12.3%) | 2.20 | <.0.0001 |
|  |  |  |  |  |  | (1.75-2.80) |  |
|  |  | **Ischemia-driven target-vessel revascularization** | | 103 (11.7%) | 64 (7.5%) | 1.59 | 0.003 |
|  |  |  |  |  |  | (1.16-2.18) |  |
|  | **Any coronary revascularization** | | | 255 (31.2%) | 113 (13.4%) | 2.39 | <.0.0001 |
|  |  |  |  |  |  | (1.92-2.99) |  |
|  |  | **Ischemia-driven any coronary revascularization** | | 120 (14.4%) | 71 (8.0%) | 1.68 | 0.0004 |
|  |  |  |  |  |  | (1.26-2.27) |  |
|  | **A composite of death, MI, stroke, or any coronary revascularization** | | | 426 (48.9%) | 281 (29.8%) | 1.67 | <.0.0001 |
|  |  |  |  |  |  | (1.44-1.95) |  |
